# SNPs in stress-responsive rice genes: validation, genotyping, functional relevance and population structure

**DOI:** 10.1186/1471-2164-13-426

**Published:** 2012-08-25

**Authors:** Swarup K Parida, Mitali Mukerji, Ashok K Singh, Nagendra K Singh, Trilochan Mohapatra

**Affiliations:** 1 National Research Centre on Plant Biotechnology, Indian Agricultural Research Institute, New Delhi, 110012, India; 2 Institute of Genomics and Integrative Biology, Delhi, 110007, India; 3 Division of Genetics, Indian Agricultural Research Institute, New Delhi, 110012, India; 4 Present Address: Central Rice Research Institute, Cuttack, 753 006, Odisha, India

**Keywords:** Illumina GoldenGate assay, Population structure, Rice, SNPs, Single nucleotide polymorphisms, Stress-responsive genes

## Abstract

**Background:**

Single nucleotide polymorphism (SNP) validation and large-scale genotyping are required to maximize the use of DNA sequence variation and determine the functional relevance of candidate genes for complex stress tolerance traits through genetic association in rice. We used the bead array platform-based Illumina GoldenGate assay to validate and genotype SNPs in a select set of stress-responsive genes to understand their functional relevance and study the population structure in rice.

**Results:**

Of the 384 putative SNPs assayed, we successfully validated and genotyped 362 (94.3%). Of these 325 (84.6%) showed polymorphism among the 91 rice genotypes examined. Physical distribution, degree of allele sharing, admixtures and introgression, and amino acid replacement of SNPs in 263 abiotic and 62 biotic stress-responsive genes provided clues for identification and targeted mapping of trait-associated genomic regions. We assessed the functional and adaptive significance of validated SNPs in a set of contrasting drought tolerant upland and sensitive lowland rice genotypes by correlating their allelic variation with amino acid sequence alterations in catalytic domains and three-dimensional secondary protein structure encoded by stress-responsive genes. We found a strong genetic association among SNPs in the nine stress-responsive genes with upland and lowland ecological adaptation. Higher nucleotide diversity was observed in *indica* accessions compared with other rice sub-populations based on different population genetic parameters. The inferred ancestry of 16% among rice genotypes was derived from admixed populations with the maximum between upland *aus* and wild *Oryza* species.

**Conclusions:**

SNPs validated in biotic and abiotic stress-responsive rice genes can be used in association analyses to identify candidate genes and develop functional markers for stress tolerance in rice.

## Background

Single nucleotide polymorphisms (SNPs) represent a robust class of molecular markers [1]. SNP markers have gained considerable importance in plant genetics and breeding because of their excellent genetic attributes and suitability for genetic diversity analysis and evolutionary relationships [2], understanding of population substructure [3-5], detection of genome-wide linkage disequilibrium [6,7], and association mapping of genes controlling complex phenotypic traits [8]. Detection and assay of SNPs are amenable to automation and thus useful for high-throughput genotyping [1].

The complete and high-quality sequence of the rice genome [9] has provided a genome-wide SNP resource comprising 5.41 million loci polymorphic between the two major cultivated rice subspecies, *indica* (93–11) and *japonica* (Nipponbare) [10,11]. This SNP resource is freely accessible at the National Center for Biotechnology Information (NCBI) SNP database (NCBI db SNP build 132) as “reference SNPs (rsSNPs)” with detailed annotation in the rice genome. Alternatively, the availability of genomic and expressed sequence tag sequences of multiple rice genotypes in the public domain has enabled identification of SNPs *in silico*[12-15]. However, efforts to validate the identified SNPs are limited, which is affecting large-scale genotyping applications in this important crop. An accurate and highly multiplexed SNP genotyping assay is thus required to use the vast SNP resource (discovered *in silico* and available in the public domain) in rice for high-throughput genetic analysis [16]. In conjunction with validation, SNP genotyping of large sets of the diverse rice germplasm (including landraces, modern cultivars and wild relatives) would enable associating natural genetic variations with polymorphisms caused by selection, population history and breeding system [17]. SNP genotyping, particularly in regulatory candidate genes for complex traits such as abiotic stress tolerance and their large-scale validation using a diverse rice germplasm panel, would be very significant for identifying novel genes and alleles possibly influenced by phenotypic selection during crop domestication that may account for rich trait diversity available in the germplasm [18]. In recent years, transcriptome profiling using rice microarrays [19] and next-generation sequencing [20] has led to identification of a large number of candidate genes for stress tolerance [21]. However, functional validation of such a large set of candidate genes in transgenics is not feasible. Candidate gene SNPs and their regulatory sequences can be used to establish a relationship between them and target stress tolerance traits by genetic association. Therefore, genotyping a diverse germplasm set using high-throughput genotyping assays is of particular interest for rice breeders to discover functionally relevant genes and new alleles [22].

Several SNP genotyping assays that were developed in the past have had varying success. These assays used four basic principles: (i) hybridization with allele-specific oligonucleotide probes [23], (ii) oligonucleotide ligation [24], (iii) single nucleotide extension [25], and (iv) enzymatic cleavage [26]. In recent years it has become feasible to simultaneously genotype large number of SNPs in a single assay due to innovative combination of miniaturized array platforms with high level of assay multiplexing and scalable automation [27]. Among these, the GoldenGate genotyping assay (Illumina, San Diego, CA) [28] has been widely leveraged to validate the large number of SNPs in many crops such as barley [29,30], maize [31,32], *Aegilops*[33], soybean [34,35], wheat [36,37], white and black spruce [38] and poplar [39]. Recently, multiplex panels of 1,536 SNPs discovered through whole genome resequencing [40] have been validated using the GoldenGate assay in rice [41,42]. Considering the greater multiplexing and high-throughput SNP genotyping potential of the GoldenGate assay, it would be relevant to utilize this technology for large-scale SNP validation and genotyping in stress-responsive rice genes using a diverse germplasm set. This in turn would help develop a gene-based SNP panel for defining population genetic structure, as well as diversity and differentiation between rice populations particularly with regard to different ecological habitat adaptation. It would further accelerate the identification of robust, functionally relevant rice genes for complex stress tolerance traits through genome-wide and candidate gene-based association analyses.

We undertook this study to: validate and genotype SNPs in a set of biotic and abiotic stress-responsive genes on a diverse rice genotype panel using bead-array based Illumina GoldenGate assay; derive the functional significance of such SNPs in terms of their evolutionary and adaptive advantages for stress tolerance; and determine the population structure in rice.

## Results

### Reproducibility, genotype call rate and success rate of GoldenGate assay

**Figure 1:**
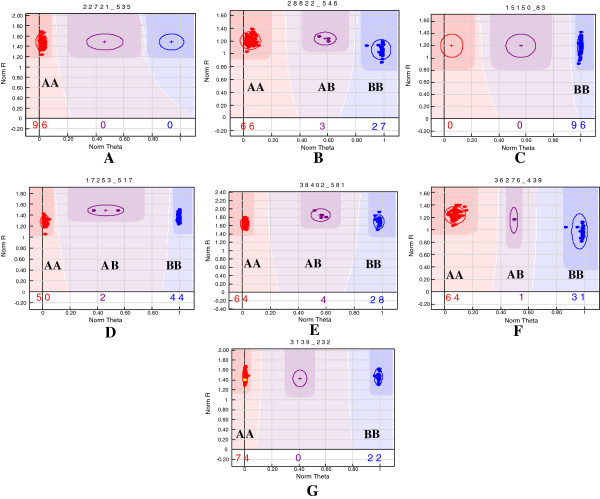
**Examples of seven SNP loci validated by the Illumina GoldenGate genotyping assay showing homozygous and heterozygous cluster separation for 91 rice genotypes based on plotting of normalized R [sum of intensities of the two channels (Cy3 and Cy5)] on the y axis vs. normalized theta [(2/π)Tan**^**-1**^**(Cy5/Cy3)] on the x axis.** A normalized theta value nearest to 0 is homozygous for allele A (*japonica* “Nipponbare” type), a theta value nearest to 0.5 is heterozygote AB and a theta value nearest to 1 is homozygous for allele B (*indica* “93–11” type). Two graphs demonstrate the monomorphic SNP loci (plots **A** and **C**), while five others (plots **B**, **D**, **E**, **F** and **G**) show polymorphic SNP loci with clear separation between the three genotypic classes.

All 384 SNP loci selected for arraying had SNP designability rank scores equal to or higher than 0.70 and thus were useful for genotyping using the GoldenGate assay. Genotyping assay reproducibility was 100% as evaluated using four samples as biological replicates — one each representing *indica*, aromatic, *japonica* and *aus*/wild rice groups. Of the 384 SNP loci, 362 (94%) (minimum GenTrain cut-off score of ≥0.25) could be genotyped successfully on all 91 genotypes. At this GenTrain cut-off score, the remaining 22 (6%) loci did not yield any genotype calls and were thus rejected. Distinct cluster separation was observed at ≥0.3 GenCall and ≥0.25 GenTrain cut-off scores across the 362 SNP loci. Genotype polar coordinate plots [normalized sum of intensities of two channels (Cy3 and Cy5) as y axis vs. normalized theta {(2/π)Tan^-1^(Cy5/Cy3)} as x axis] of these loci were used to classify the 91 rice genotypes into one of three clusters: (i) homozygous AA (*japonica* “Nipponbare”), (ii) homozygous BB (*indica* “93–11”), and (iii) heterozygous AB (Figure 1).

Based on minimum 0.3 GenCall and 0.25 GenTrain cut-off scores optimized in this study, 787 (2%) of 32,942 genotype calls were identified as missing data. The remaining 32,155 yielded successful genotype calls giving a high average call rate of 98% per valid SNP for rice genotypes. When we increased the Gen-Train score cut-off value to ≥0.4, the average missing data rate per successful SNP in rice genotypes decreased to ≤0.5%. Three hundred twenty-five (90%) of the 362 SNP loci, which produced 29,575 genotype calls, showed polymorphism (see Additional file 1) while the remaining 37 (10%) were monomorphic. Excluding the monomorphic loci, the overall genotyping success rate or SNP conversion rate of the GoldenGate assay was 85% across 91 diverse rice genotypes using “rice OPA-1”. Three hundred twenty-five SNP loci validated in the stress-responsive genes were physically mapped (MSU Rice Genome Annotation Project, release 6.1) on 12 rice chromosomes (see Additional file 2). The majority of these SNPs (288, 89%) were present in the coding regions and the rest (37, 11%) in the 5’untranslated regions of the selected genes. The polymorphism rate at these SNP loci across chromosomes varied from 75% in chromosome 10 to 100% in chromosome 3 with an average of 85% (see Additional file 2). Of the 325 polymorphic SNP loci, 263 (81%) and 62 (19%) were validated in abiotic and biotic stress-responsive rice genes, respectively.

### SNP validation in diverse Oryza sativa and wild species genotypes

**Figure 2:**
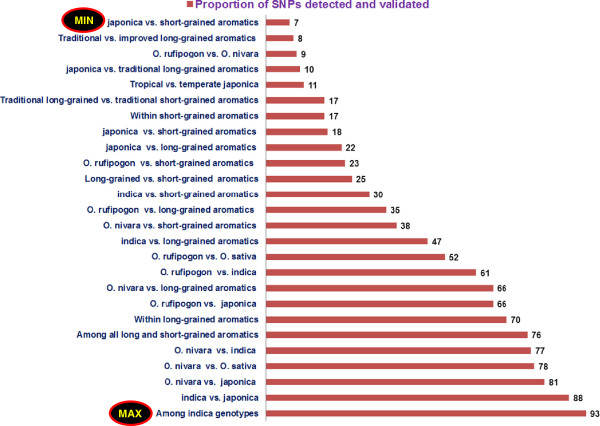
**Proportion of SNPs detected and validated in *O. sativa *and wild species using the GoldenGate genotyping assay.** Maximum SNPs were validated among *indica* genotypes (93%) followed between *indica* and *japonica* (88%), and minimum between *japonica* and short-grained aromatics (7%).

A total of 325 SNPs, consisting of 207 (64%) transitions and 118 (36%) transversions, were validated using the “rice OPA-1” high-throughput bead array-based assay. The higher frequency of transitions versus transversions in validated SNPs was comparable to the *in silico* estimate (63%). SNP loci that differentiated any two individual genotypes belonging to two different groups were considered polymorphic between the groups to which they belonged. Based on this criterion, 254 (78%) were polymorphic between *O. nivara* and *O. sativa*, 168 (52%) between *O. rufipogon* and *O. sativa*, and 28 (9%) between *O. rufipogon* and *O. nivara* (Figure 2). Two hundred sixty-two (81%) SNPs were validated between*O. nivara* and *japonica*, 249 (77%) between *O. nivara* and *indica*, 215 (66%) between *O. nivara* and long-grained aromatics, and 76 (23%) SNPs between *O. rufipogon* and short-grained aromatics. Within *O. sativa*, the most polymorphism was observed between *indica* and *japonica* (285 SNP loci, 88%) and the least was between *japonica* and short-grained aromatics (59 loci, 18%) (Figure 2). Among the aromatics, 82 (25%) SNPs were validated between long and short-grained accessions, while 26 (8%) were validated between traditional and improved long-grained aromatics. The polymorphic SNP loci frequency was highest within *indica* (93%, 302 SNPs) and lowest between the two *japonica* genotypes (11%, 37) (Figure 2).

### SNP characteristics and functional significance

**Figure 3:**
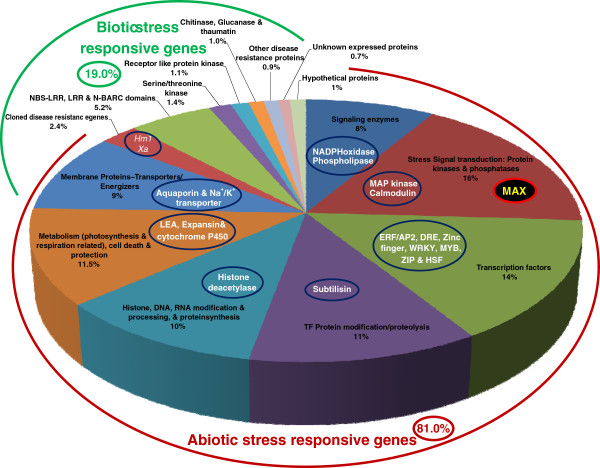
**Functional annotation of 263 abiotic stress-responsive rice genes with validated SNPs.** Most genes belong to signal transduction pathway-related gene families like calcium dependent and/mitogen activated protein kinases (16%) while the fewest number of genes encode signaling enzymes such as nicotinamide adenine dinucleotide phosphate (NADPH)-oxidase and phospholipase (8%). Examples of important genes in each category are highlighted.

The functional annotation of 263 abiotic stress-responsive rice genes with SNPs revealed maximum correspondence to stress signal-transduction pathway gene families (16%) followed by transcription factors (Figure 3). The average ratio of non-synonymous to synonymous SNPs in the coding regions of these genes was 0.98, which is lower than that estimated (1.4) for all 325 polymorphic genes. In contrast, SNPs in the coding regions of 62 known and candidate disease resistance genes exhibited a higher average ratio (1.6) of non-synonymous to synonymous substitutions. A total of 40 (12.3%) SNP loci in the abiotic (16 loci, 6%) and biotic (24 loci, 38.7%) stress-responsive rice genes resulted in non-synonymous substitutions and thus are expected to have affected the encoded proteins. This included SNPs in nine abiotic stress-related genes that differentiated 22 upland *indica* and two genotypes of wild species from 30 lowland *indica* and 19 aromatic rice genotypes. Four of these SNPs — found in a MYB family transcription factor, a sucrose transporter, a calcium dependent protein kinase and a WRKY family transcription factor (Table 1 and see Additional file 1) — resulted in missense substitutions and amino acid replacements (E to V, R to H, V to A and Y to S, respectively). The remaining five SNPs validated in genes encoding cytochrome P450, heat shock protein, pyruvate kinase, translation elongation factor and soluble acid invertase, resulted in introduction of premature termination codons ochre, opal and amber. Although biotic stress-responsive genes had more non-synonymous substitutions, none of the corresponding SNPs differentiated upland *indica* from lowland *indica* genotypes. When we considered specific cases, non-synonymous SNP loci in two disease resistance-like genes encoding NBS-LRR (LOC_Os04g54780) and NB-ARC domains (LOC_Os04g30930) differentiated the bacterial leaf blight (BLB) resistant *indica* rice genotypes Aditya from BLB susceptible aromatic rice genotypes. Another two SNP loci found in genes containing serine threonine protein kinase (LOC_Os09g37949) and leucine-rich repeat (LOC_Os04g19750) domains differentiated eight rice blast resistant *indica* genotypes (Rasi, Khandagiri, Birsadhan, Nilagiri, Subhadra, Samanta, Pathara and Badami) from the susceptible *indica* genotypes (see Additional file 3).

**Table 1:** Non-synonymous SNPs in stress-responsive rice genes that differentiated upland genotypes from lowland genotypes of rice

**Functional classifications**	**Stress-responsive rice genes**	**Codon substitutions**	**Amino acid replacement**	**Types of mutation**	**Possible causes of altered secondary protein structure and functions**
Transcription factors	WRKY family transcription factor	TAC to TCC	Tyrosine to Serine	Missense substitution	Conserved WRKY DNA binding domains and differential sequence-specific DNA binding affinity
	MYB family transcription factor	GAG to GTG	Glutamic acid to Valine	Missense substitution	Conserved N-terminal tryptophan clustered Myb DNA binding domains and differential DNA binding affinity
	Heat Shock factor	CGA to TGA	Glycine to Opal	Nonsense/Introduction of premature termination codon	Conserved C-terminal basic zipper signal sensing domains that senses external signals and transmit to transcription DNA binding domains of same protein
Metabolism, photosynthesis and protein synthesis related	Sucrose transporter	CGC to CAC	Arginine to Histidine	Missense substitution	Transmembrane beta-stranded domain affects apoplastic sucrose transportation by protonophores/cation symporter activity
	Pyruvate kinase	CAA to TAA	Glutamine to Ochre	Nonsense/Introduction of premature termination codon	Enzyme regulatory active catalytic domain binding site with substrate (phosphoenol pyruvate) and Mn^2+^ ligand complexes affects allosteric mechanism of glycolysis
	Soluble acid invertase	CAA to TAA	Glutamine to Ochre	Nonsense/Introduction of premature termination codon	Small conserved amino acid sequence in active domain binding site with substrate and Na^+^ ligand complexes regulates sucrose to hexose ratio in vacuole
	Cytochrome P450	CAG to TAG	Glutamine to Amber	Nonsense/Introduction of premature termination codon	Conserved cysteine residues active domain binding site with Fe^3+^ ligand complexes that regulates multi-component electron transfer chains in photosynthesis
	Elongation factor	CAG to TAG	Glutamine to Amber	Nonsense/Introduction of premature termination codon	A GTP-binding catalytic domain with Mg^2+^ ligand binding complexes affects binding of aminoacyl tRNA
Stress signal transduction	Calcium dependent Protein kinase	GTC to GCC	Valine to Alanine	Nonsense/Introduction of premature termination codon	Glycine-rich ATP conserved catalytic domain and peptide-substrate and Ca^2+^ ligand binding site affects phosphorylation
Resistance gene analogue (RGA)	Leucine-rich repeat domain	GTC to GCC	Valine to Alanine	Missense substitution	Dimerization of functional domain and altered ligand binding site
	NBS-LRR disease resistance	TCC to TAC	Serine to Tyrosine	Missense substitution	Dimerization of functional domain and altered ligand binding site
	Serine/threonine protein kinase	CAG to CGG	Glutamine to Arginine	Missense substitution	Conserved functional domain and altered ligand binding site affects phosphorylation
	NB-ARC domain	GAT to GGT	Aspartic acid to Glycine	Missense substitution	Conserved functional domain and altered ligand binding site

**Figure 4:**
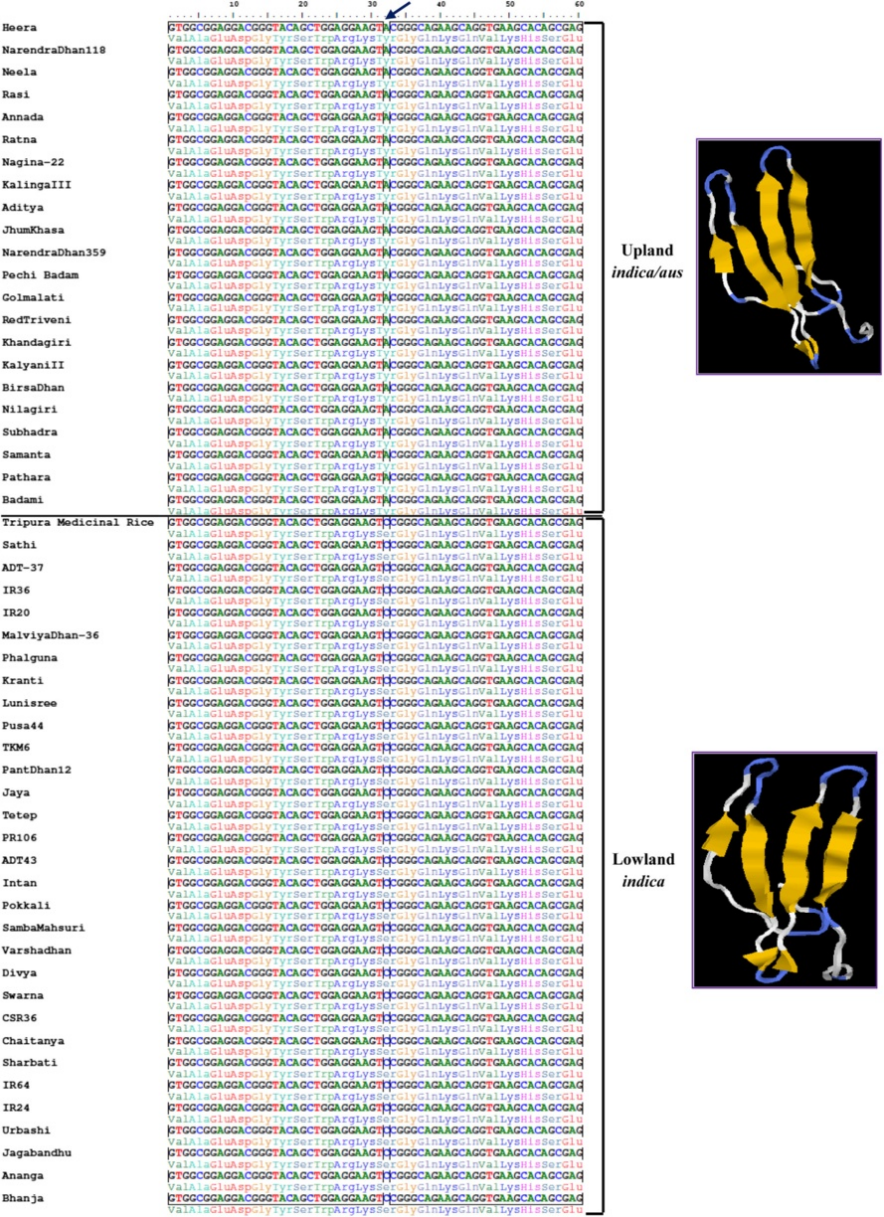
**Nucleotide sequence alignment and predicted protein structure depicting the functional relevance of non-synonymous SNP in abiotic stress-responsive WRKY gene.** Non-synonymous SNP validated in WRKY family (*OsWRKY35V2*) transcription factor gene (LOC_Os04g39570) showing differentiation between 22 drought tolerant upland *indica*/*aus* and 30 drought sensitive lowland rice genotype groups. Missense transversional substitution of the second nucleotide in the triplet codon of upland *indica*/*aus* genotypes coding for amino acid in the conserved DNA binding domain of WRKY by another nucleotide resulted in the formation of new triplet codon encoding for novel amino acid in lowland *indica* group. The substitution mutation was predicted to alter three dimensional secondary structure of protein including the DNA binding domain in WRKY which may produce functionally different protein between upland and lowland genotypes. Missense non-synonymous SNP site, different protein structure and DNA binding domain are highlighted.

**Figure 5:**
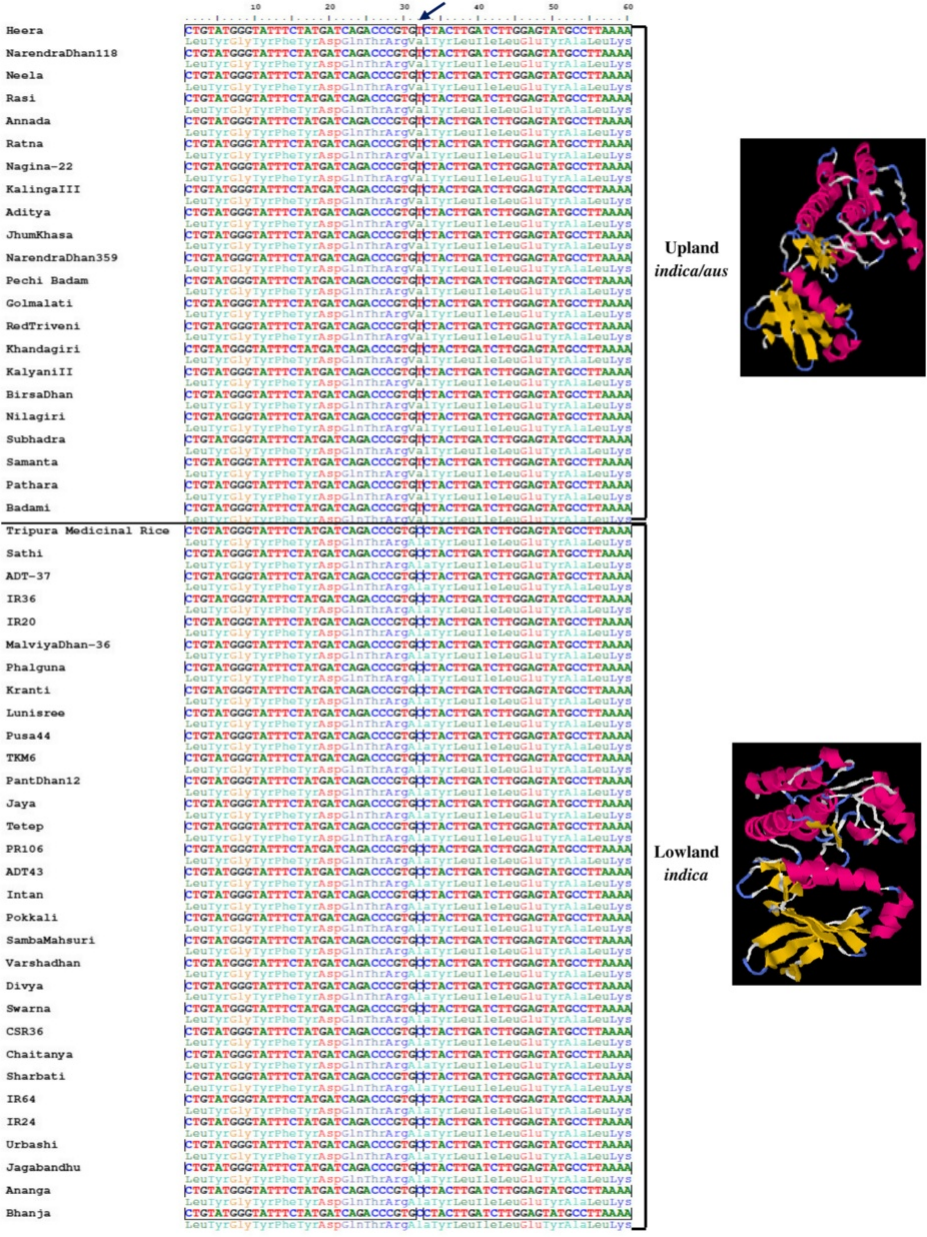
**Nucleotide sequence alignment and predicted protein structure depicting the functional relevance of non-synonymous SNP in abiotic stress-responsive protein kinase gene.** Non-synonymous SNP validated in calcium dependent protein kinase gene (LOC_Os01g09580) showing differentiation between 22 drought tolerant upland *indica*/*aus* and 30 drought sensitive lowland rice genotype groups. Missense transversional substitution of the second nucleotide in the triplet codon of upland *indica*/*aus* genotypes coding for amino acid in the glycine-rich ATP conserved catalytic binding domain of protein kinase by another nucleotide resulted in the formation of new triplet codon for novel amino acid in lowland *indica* group. The substitution mutation predicted to alter three dimensional secondary structures of protein including peptide-substrate and Ca^2+^ ligand-binding site of catalytic domain in protein kinase which may produce functionally different proteins between upland and lowland genotypes. Missense non-synonymous SNP site, different protein structure and DNA binding domain are highlighted.

We performed an *in silico* analysis of the structure predicted from amino acid sequences of functional domains to understand the possible biological significance of non-synonymous SNP loci in the functional domains carrying nine abiotic stress-responsive genes. Results revealed alteration in secondary structure of encoded proteins due to missense and nonsense mutations in the functional domains encoded by the nine abiotic stress-responsive genes that differentiated the upland and lowland genotypes from each other. The variant form in contrast to native form of proteins encoded by three transcription factor genes — WRKY, MYB and heat shock factor (HSF) — showed amino acid sequence change in DNA binding/signal sensing domains (Table 1). For example, the upland rice genotypes had tyrosine (Y) in the conserved DNA binding domain (WRKYGQK) of protein encoded by WRKY transcription factor (*OsWRKY35V2*), which was found substituted by serine (S) in the lowland genotypes. This possibly resulted in decreased binding affinities of WRKY domains to invariant ‘TGAC’ core of W box. It is likely that substitution mutations in WRKY impart differential hydrophobic interactions and beta sheet’s stability thus creating a varied zinc finger and DNA binding protein structure between drought tolerant upland and sensitive lowland rice genotypes (Figure 4). However, the remaining six genes (belonging to metabolism, photosynthesis, protein synthesis and stress signal transduction pathway groups) showed variations in the active allosteric regulatory catalytic domain of proteins that bind with substrate and ligand complex (Table 1). For example, missense substitution of amino acid valine in the glycine-rich ATP conserved catalytic domain of protein kinase gene in upland rice genotypes with alanine in lowland genotypes resulted in an altered secondary protein structure. This may affect the peptide-substrate and Ca^2+^ ligand-binding sites of catalytic domain (Figure 5) and phosphorylation activity of the regulated gene. Similarly, in four disease resistance genes, missense substitutions of amino acid in the functional domains altered the secondary protein structure that possibly affected dimerization domains and ligand-binding sites.

### Analysis of molecular diversity, population structure and genetic association

The polymorphism information content (PIC) based on SNPs in the stress-related rice genes varied widely across the 89 *O. sativa* genotypes. Higher nucleotide diversity within *indica* (PIC = 0.46) compared with long (0.40) and short (0.32) grained aromatics, *japonica* (0.2841) and *aus*/wild species (0.25) was evident. We found the average nucleotide diversity (PIC =0.44) across 263 SNP loci in candidate abiotic stress response genes to be higher specifically in upland *indica* genotypes than that observed in the 62 candidate biotic stress-related genes (PIC = 0.27).

**Figure 6:**
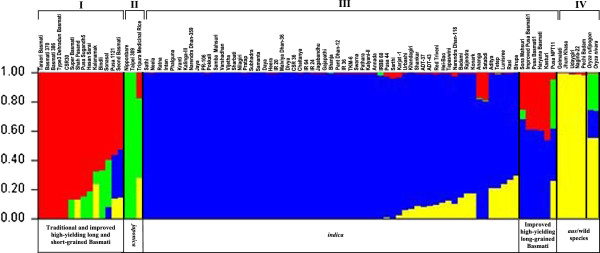
**Population structure (K = 4) of accessions used in for SNP validation (n = 91).** The rice genotypes were assigned to four distinct groups namely *indica*, aromatic, *japonica* and *aus*/wild using 325 SNP loci. Vertical bars along the horizontal axis represent rice genotypes classified into K color segments according to the estimated membership fraction of genotypes in each K cluster.

Population genetic structure analysis among the 91 genotypes using the Bayesian clustering algorithm of STRUCTURE with varying K (number of sub-populations) levels revealed that at K value of 2 all genotypes were classified into two distinct sub-populations, *indica* and *japonica*/aromatic (see Additional file 4). When the K value was increased to 3, aromatic and *japonica* groups emerged as independent sub-populations and clustered separately from *indica* sub-population. The best replicate giving maximum log likelihood values was obtained when K was set at four (Figure 6). At this K value, the genotypes were grouped into four sub-populations that corresponded well with their expected taxonomic and pedigree relationships, which was comparable to the clustering pattern as depicted by the neighbor-joining tree based on pair-wise genetic distances. However, when K value was increased to 5, no additional cluster with high-resolution population structure was obtained, and relationships among genotypes as observed at K = 4 remained intact (see Additional file 4). The rice genotypes used in our study were thus classified into four distinct sub-populations: group I consisting of 11 genotypes of traditional and improved high-yielding long and three short-grained aromatics; group II comprising one genotype each of tropical and temperate *japonica* and Tripura Medicinal Rice; group III with 61 *indica* type, five improved high-yielding long-grained aromatics and Pusa NPT11; and group IV consisting of five *indica* (possibly upland *aus* type), two wild species *O. rufipogon* and *O. nivara*. Molecular genetic variation among and within these four sub-populations (determined by pair-wise estimate of divergence (mean F_ST_) and genetic distance (D_ij_) based on 325 SNP loci), revealed a wide quantitative genetic differentiation in *O. sativa* (F_ST_ 0.20 to 0.92 with an average of 0.46; D_ij_ 0 to 0.0235 with mean 0.0056) (see Additional file 5). Pair-wise F_ST_ values among these four sub-populations indicated maximum divergence between *indica* and *japonica* (F_ST_ = 0.89) and minimum between aromatics and *japonica* (0.28) (see Additional file 5).

**Figure 7:**
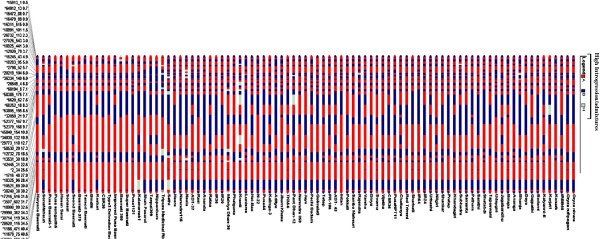
**Genomic constitution of 91 rice genotypes based on introgression of *indica *and *japonica *alleles for rice chromosome 1 determined using 43 SNP loci validated in stress-responsive rice genes.** The discriminated allele types A (homozygous for allele A, *japonica* “Nipponbare” type), B (homozygous for allele B, *indica* “93–11” type) and H (heterozygous AB) are marked in red, blue and grey colors, respectively. Maximum recombination and introgression was observed in the short-arm of this chromosome.

All 91 genotypes belonged to a single population in which more than 80% of their inferred ancestry was derived from one of the model-based populations and more than 16% contained admixed populations defined as “admix” (see Additional file 6). An 18% admixture was observed between the five *indica* (possibly upland *aus* type) and the wild *Oryza* species while between *japonica* and long-grained aromatics it was ~ 8%. Tripura Medicinal Rice had maximum (54%) admixtures of *japonica* followed by that of *indica* (18%), traditional Basmati (15%), short-grained aromatics (5%) and the wild (2%) species (Figure 6). Genomic constitution analysis of rice genotypes based on chromosome-wise physical distribution of variant SNP loci and allele sharing between *indica* and *japonica* revealed maximum introgression of *japonica* in chromosome 12 (average 63%) followed by chromosomes 7 (59%) and 1 (57%); chromosome 6 contained maximum introgression (68%) of *indica*. In contrast there was equal sharing (~ 49%) of genomic regions in chromosomes 2 and 8 of *japonica* and *indica*. Introgression frequency (based on the number of recombination events) was the most in chromosome 1 (particularly the short arm; Figure 7 and see Additional file 7) and fewest in chromosome 3 (see Additional file 8). Overall, maximum introgression between *indica* and *japonica* was observed in Tripura Medicinal Rice (see Additional file 9), which also exhibited the highest degree of heterozygosity (21%). The allele sharing map (Figure 7 and see Additional file 7) for 12 chromosomes of the 91 genotypes identified 10 large introgression regions carrying 40 non-synonymous SNP loci in the biotic and abiotic stress-responsive genes that are expected to be under artificial selection pressure. Interestingly, one such introgression region on chromosome 4 and another on chromosome 9, each containing six SNP loci in the abiotic stress-responsive genes, differentiated drought tolerant upland *indica* and wild rice genotypes from lowland *indica* and aromatic rice genotypes used in our study.

Genetic association analysis using genotyping data of 325 SNPs in stress-responsive genes and phenotypic information of 91 rice genotypes belonging to different ecosystems revealed 18 non-synonymous SNPs associated with ecological adaptation at *P* < 0.05 and R^2^ ≥ 0.90 (Table 2). Significantly of these, nine SNPs (discriminating all upland *indica* and wild rice genotypes from lowland rice genotypes) showed strong association with ecological adaptation at *P* < 0.01 and R^2^ = 1.00.

**Table 2:** Non-synonymous SNPs in stress-responsive rice genes showing significant association with ecological differentiation of rice genotypes

**Stress-responsive rice genes**	**MSU locus IDs**	**Major allele in upland rice genotypes**	**Minor allele in lowland rice genotypes**	**P value**	**R**^**2**^**value**
WRKY family transcription factor	LOC_Os04g39570	A	C	0.0012	1.00
Calcium dependent protein kinase	LOC_Os01g09580	T	C	0.0027	1.00
MYB family transcription factor	LOC_Os02g02370	A	T	0.0031	1.00
Heat Shock factor	LOC_Os02g29100	C	T	0.0032	1.00
Sucrose transporter	LOC_Os10g26470	G	A	0.0043	1.00
Pyruvate kinase	LOC_Os04g58110	C	T	0.0047	1.00
Soluble acid invertase	LOC_Os09g06790	C	T	0.0035	1.00
Cytochrome P450	LOC_Os04g40470	C	T	0.0030	1.00
Elongation factor	LOC_Os06g37440	C	T	0.0050	1.00
Leucine-rich repeat domain	LOC_Os04g19750	T	C	0.0190	0.94
NBS-LRR disease resistance	LOC_Os04g54780	C	A	0.0290	0.91
Serine/threonine protein kinase	LOC_Os09g37949	A	G	0.0470	0.90
NB-ARC domain	LOC_Os04g30930	A	G	0.0500	0.90
40S ribosomal protein S24	LOC_Os06g40650	A	G	0.0290	0.90
AP2-like ethylene responsive transcription factor	LOC_Os02g40070	C	G	0.0280	0.90
ABC transporter family protein	LOC_Os11g05700	A	C	0.0340	0.92
Zinc finger C3HC4 type family protein	LOC_Os11g36560	A	G	0.0460	0.90
OsWAK receptor-like protein kinase	LOC_Os12g30460	G	T	0.0380	0.91

## Discussion

### Genotyping success rate of GoldenGate assay and validation of SNPs in stress-responsive rice genes

Illumina GoldenGate platform has shown exceptional performance with regard to throughput, reproducibility, genotype call rate and assay development success rate among several SNP genotyping assays involving human and a few plant species, [29,34,43]. In our study, 384 SNP loci with a high predetermined SNP designability rank score of ≥0.70 were selected to develop a “rice OPA-1” array for use with the GoldenGate platform. The SNPs were chosen based on their presence in biotic or abiotic stress-responsive genes (one SNP per gene) and distribution over 12 rice chromosomes. Genotyping of four biological replicates of DNA samples gave 100% reproducibility that suggested robustness of the GoldenGate assay for SNP validation and genotyping in rice. The average genotype call rate of 98% per valid SNP at ≥0.25 GenTrain cut-off score is comparable to that obtained earlier for white and black spruce (99%) [38] and human (100%) [43,44], using the same stringent criteria for SNP genotyping using the GoldenGate assay. About 2% genotype calls were identified as missing data at ≥0.25 GenTrain score, which is comparable to previous reports from other plant species [34,38].

There was a distinct separation of two homozygous classes expected in true breeding inbred lines for 362 SNP loci at ≥0.3 GenCall and ≥0.25 GenTrain scores used in this study. A high proportion of homozygous (99.3%) SNP calls was expected in view of the highly self-pollinating nature of cultivated rice. With 325 validated SNP loci, the overall genotyping success rate/SNP conversion rate was 85%, which is comparable to the estimate obtained (84%) earlier with GoldenGate genotyping assay in rice [42]. The observed success rate is different from that in other studies (varied from 77 to 91%) [29,32,38], which is expected due to species differences, genotypes and SNP loci used. The lower SNP conversion rate in our study could be due to errors in low quality genome sequence of *indica* genotype 93–11 contributing to false SNPs at 37 of 362 loci for which genotype calls were made. With the recent availability of high quality genomic sequence from multiple genotypes [9,40-42], miscalled SNPs due to poor genomic sequence should not be a concern and eliminating possible SNP loci lacking necessary flanking sequence specificity for successful conversion should be much easier. Overall, our study revealed that a highly multiplexed universal custom SNP array “rice OPA-1” designed for Illumina GoldenGate assay targeting a set of stress-responsive genes was efficient enough to rapidly genotype rice accessions with high precision and success rate.

The SNPs validated in coding (89%) and non-coding (11%) sequence regions of stress-responsive genes have the potential to be used as candidate gene-based functional markers for various stress tolerance traits in rice. Sixty-eight percent of the SNP loci that were found to be polymorphic between *indica* and *japonica in silico* contained exactly the same SNP alleles between 68 *indica* and two *japonica* rice genotypes with ± 0.15 to ± 0.2% low-end error rate. This is higher than the efficiency range reported (46 to 55%) earlier based on direct amplicon sequencing [10,12]. The transition and transversion frequencies obtained in our study are comparable (63%) to that observed *in silico* between *indica* and *japonica*. The relatively higher (70%) frequency of transition substitutions validated between *indica* and *japonica* than among *indica*, aromatic and wild species groups (60–65%), agreed well with earlier genome-wide SNP discovery studies involving *indica* and *japonica* rice subspecies [9-12,14].

The allele sharing map constructed separately for 12 chromosomes of 91 rice genotypes identified two large introgression regions carrying each of six SNP loci in the abiotic stress-responsive genes that differentiated the known stress tolerant upland *indica* and wild rice accessions from the sensitive lowland *indica* and aromatic rice genotypes. It would be interesting to use this SNP subset in adaptive trait-specific association analysis. The high and low introgression rates and admixtures revealed by variant SNP loci across chromosomes of different domesticated *O. sativa* and wild species genotype groups in our study reflect differential SNP allele sharing between respective gene pools of *O. sativa* and wild species [10,40,45,46].

### Functional and adaptive significance of SNPs validated in stress-responsive rice genes

Higher average ratio of non-synonymous to synonymous substitutions in the coding regions of biotic stress-responsive rice genes compared to abiotic stress-related genes was possibly due to diverse nature of plant disease resistance proteins that evolved as a result of pathogen pressure [47,48]. Detection of nine non-synonymous SNP loci showing missense and non-sense mutations in important abiotic stress-responsive rice genes (WRKY and MYB family transcription factors, calcium dependent protein kinase, heat shock factor, sucrose transporter, pyruvate kinase and soluble acid invertase, cytochrome P450 and elongation factor) that differentiated the contrasting known drought-tolerant upland *indica* rice genotypes from sensitive lowland genotypes suggests the functional significance of SNPs in these genes [49]. Further, missense non-synonymous SNP loci in the biotic stress response-related genes encoding NBS-LRR and serine/threonine protein kinase domains that differentiated selected upland *indica* rice genotypes from lowland genotypes may also be relevant for their differential reaction to diseases such as blast and bacterial blight.

Correlation between non-synonymous SNPs in functional domains encoded by nine abiotic stress-responsive genes with alteration in predicted secondary protein structure and catalytic domain binding sites suggests functional relevance of such SNPs. This was further evident from the high-degree association of these nine non-synonymous SNPs in genes with upland and lowland adaptive differentiation. For example, the functional significance of one such SNP, showing missense substitution in the conserved DNA binding domain of protein encoded by WRKY transcription factor gene was assessed by correlating its altered secondary protein structure with DNA binding and transcriptional activity. Differential DNA binding selectivity of WRKY transcription factor towards consensus ‘TGAC’ core of W box due to non-synonymous substitutions and its correlation with differential sensitivity to various abiotic stresses, particularly drought, have been reported previously for *Arabidopsis thaliana*, *Brassica napus*, tobacco and rice [50-53]. The evolutionary and adaptive advantages of such non-synonymous SNPs in genes that affect the structure and function of encoded proteins to generate favorable alleles for mitigating environmental stress impact under high selection pressure through modulation of mutation in these loci have been reported in eukaryotes [54]. For example, in *Pinus* the non-synonymous amino acid substitution in the protein coding regions of drought-responsive genes have provided greater adaptability to various abiotic stresses [55]. Similarly, the adaptive advantage of non-synonymous SNP loci in bacterial blight resistance gene affecting the dimerization and active ligand binding sites of proteins has been demonstrated [56]. Understanding the adaptive significance of such mutations in genes needs further experimentation using a larger set of contrasting genotypes.

### Understanding diversity pattern and population genetic structure in rice

The population structure based on 325 polymorphic SNP loci identified four major model-based genetically distinct groups namely, *indica*, *japonica*, aromatics and *aus*/wild. To generate these four sub-populations, a burn-in length of 50,000 and run length of 100,000 iterations was sufficient to obtain reasonably consistent values of maximum log likelihood across 20 replicates. This observation is comparable to those based on population structure analysis using microsatellite and SNP markers [3,5,57-59]. The genetic diversity estimated among the rice sub-populations in our study was much higher than that obtained previously (0.20 to 0.42 with an average of 0.37 [58]; 0.11 to 0.72 with an average of 0.31 [7]) with microsatellite and SNP markers, but comparable (0.047 to 0.76 with an average of 0.47) to that detected in a larger set of rice genotypes using microsatellite markers [60].

The higher PIC in *indica* than in aromatic, *japonica* and *aus*/wild groups agreed well with earlier observations using microsatellite and SNP markers [4-7,57-60]. Higher nucleotide diversity in *indica* was most likely the result of strong purifying selection for specific SNP-containing genes which was evident from higher PIC across SNP loci. This could be due to the diverse rainfed/irrigated lowland, medium land and upland rice genotypes included under the *indica* group which resulted in higher number of polymorphic SNP loci detection, and higher modal nucleotide substitution and amino acid replacement than that of other domesticated *O. sativa* groups. All these observations together suggest higher allelic diversity within *indica* than aromatics, *japonica* and *aus*/wild rice genotypes. The higher nucleotide diversity and PIC in long-grained aromatics than short-grained aromatics and *aus*/wild groups was due to inclusion of traditional Basmati, which are selection products from landraces and improved high yielding Basmati developed through cross-breeding involving traditional Basmati and non-Basmati *indica* varieties [61].

Population structure analysis revealed that a set of 11 genotypes from four rice sub-populations (Tripura Medicinal Rice, five improved high-yielding long-grained Basmati and five upland *indica*/possibly *aus* type) had population admixture (>16%) with more than one genetic background, which may have resulted from their complex breeding history involving intercrossing and introgression between germplasm coupled with strong selection pressure. This was evident from clustering of five improved high-yielding long-grained Basmati within the *indica* sub-population in our study, which is expected because all improved Basmati genotypes were developed through cross-breeding involving non-aromatic *indica* and traditional Basmati germplasm [62,63]. The five *indica* types such as Nagina22 that showed about 15% admixture with *O. rufipogon* were predicted as *aus* type [40,64] under this category [65]. This observation suggests that *aus* types most likely evolved through natural cross-hybridization involving wild species, and subsequently were selected and domesticated by farmers. However, a complete understanding about the domestication and evolutionary history of these possible upland *aus* types and other *O. sativa* and wild rice sub-populations would require analysis of a greater number of well characterized and known *aus* type rice cultivars. Maximum (54%) admixtures of *japonica* in Tripura Medicinal Rice support earlier observations on possible inter-ecotype hybridization as evident from *japonica* type cytoplasm in north-eastern hilly region *indica* genotypes [66]. Introgression of different small chromosomal segments of *indica* and *japonica* into Tripura Medicinal Rice is possibly because of hybridization of male *indica* with female *japonica* followed by cross-hybridization of the resultant hybrid as female with *japonica* during domestication in north-eastern India. Greater than 18% admixture between wild and upland *indica* rice sub-populations for putative stress gene SNPs suggests likely introgression of trait-associated genomic regions from wild species into domesticated *indica* genotypes.

## Conclusions

The results have encouraging implications for use of bead array platform-based Illumina GoldenGate assay for validation and genotyping of SNPs in a specific set of stress-responsive genes for understanding their functional relevance. The results also suggest the feasibility of using SNPs as markers in identification and targeted mapping of trait-associated genomic regions for stress tolerance and further for adaptive trait-specific association analysis in rice.

## Methods

### Germplasm selection

Eighty-nine *Oryza sativa* genotypes including 68 *indica*, 19 aromatic, 1 tropical *japonica*, 1 temperate *japonica*, and two wild species (*O. rufipogon* and *O. nivara*) were selected for validation and genotyping of SNPs (see Additional file 6). The aromatic group consisted of four traditional and 12 improved high-yielding long-grained Basmati and three short-grained aromatics. The *indica* group included 22 upland and 46 medium/lowland rice genotypes (see Additional file 7). Seventy-one of these genotypes were developed through cross-breeding and the remaining (18) were selections from landraces.

### SNP selection for designing GoldenGate custom oligo pool and bead array construction

SNPs were selected from 408,898 SNPs discovered earlier by *in silico* analysis of *indica* (93–11) and *japonica* (Nipponbare) genomic sequence [10]. All SNPs were first annotated on newly released pseudomolecules (MSU Rice Genome Annotation Project, release 6.1, [67]) of 12 rice chromosomes and 500 bp genomic sequences covering SNP loci were retrieved. These SNP-containing genomic sequences were further BLAST searched [68] against the latest annotated TIGR rice genes. Five hundred sixty of these sequences showing unique BLAST hits and high degree of sequence homology with non-redundant rice genes at *E* value = 0 and bit score ≥500 that corresponded to different classes [69,70] of known disease resistance (*R*) genes/Resistance Gene Analogues and various abiotic stress-responsive genes belonging to different functional categories [19,21,71] and plant gene ontologies [72,73] were selected for further analysis. The selected genomic sequences were analyzed using the Illumina Assay Design Tool to design the custom oligo pool assay (OPA) called as “rice OPA-1”. Three hundred eighty-four SNPs, one from each stress-related gene with minimum oligo designability cut-off rank scores of ≥0.7, were then selected for synthesis of a custom Sentrix Array Matrix (SAM) by Illumina (San Diego, CA, USA).

### Generation and curation of SNP genotyping data

The GoldenGate assay [43,44] was performed in accordance with the manufacturer’s protocol for plant genomes with minor modifications as described [34,38]. The total genomic DNA isolated from fresh bulked leaf tissue of 15–20 plants per genotype was quantified and diluted to 50 ng/μl with TE (10 mM Tris–HCl, pH 8.0 and 1 mM EDTA) buffer to make single-use DNA. Allele specific oligonucleotide hybridization was carried out using 5 μl of single-use template genomic DNA (50 ng/μl) of each genotype. Four genotypes (IR64, Taraori Basmati, Nipponbare and *O. rufipogon*) were used as biological replicates to evaluate the reproducibility of the genotyping assay. Template DNA and a non-template (water) negative control were hybridized with 384 different SNP loci containing OPA and an allele-specific multiplexed primer extension and ligation reaction was performed. Following polymerase chain reaction (PCR) with a set of fluorescent dye-labeled (Cy3 and Cy5) universal primers, the labeled PCR products were hybridized onto a decoded SAM and finally analyzed using the bead array reader software module of Illumina Bead Station 500 G.

The intensity data for each SNP were normalized and cluster positions were assigned using Illumina BeadStudio Genotyping software module. The quality scores represented by GenCall and GenTrain scores were estimated for each SNP call that reflected the degree of separation between homozygous and heterozygous clusters for each SNP locus and placement of individual SNP call for each genotype within a cluster [38]. Minimum GenCall and GenTrain cut-off scores of 0.25 were used to determine valid genotypes at each SNP locus and measuring reliability of SNP detection based on distribution of genotypic classes, respectively. Different parameters of genotyping performance, such as reproducibility, genotype call rate and assay development success rate were estimated [43]. The cluster separation score provided by GenCall software module for 91 individual rice genotypes was optimized manually based on degree of separation between the two homozygous clusters as normalized θ value [(2/π) Tan^-1^ (Cy5/Cy3)], which is expected to be much more informative in plant genomes [34]. All allelic data were manually checked for errors in calling the homozygous and heterozygous clusters for each SNP locus. The placement of most reliable individual genotype calls within a distinct cluster was considered ‘successful’ and the remaining were marked as ‘null alleles’. Graphical outputs of genotyping data as heat maps and scatter plots were then generated for individual SNP locus and used for further analysis.

### Frequency of SNPs, recombination and introgression

The SNPs in stress-responsive rice genes were categorized according to nucleotide substitutions as either transitions (C/T or G/A) or transversions (C/G, A/T, C/A and T/G), and their frequency of occurrence determined individually in different rice genotype groups. The physical position of each validated SNP locus showing allelic variation was determined based on their annotations on the 12 rice chromosomes as described above. The physical locations (bp) of validated SNPs on the 12 chromosomes were used in Graphical GenoTypes [74] Version 2.0 for determining the genomic constitution of rice genotypes. SNP alleles for each locus were marked in different colors and incorporated in ascending order of physical location (bp) beginning from the short arm telomere to the long arm telomere of each rice chromosome to generate allele sharing maps of individual rice genotypes and determine the extent of recombination and introgression across chromosomes.

### Functional relevance of SNPs in stress-responsive genes

The divergence of coding sites in each of the variant SNP loci based on derived non-synonymous substitutions (degree of amino acid changes) was analyzed individually for *O. sativa* and wild rice genotype groups using the “preferred” and “unpreferred” concepts of codon usage pattern in *Oryza*[75]. Amino acid sequences encoded by the coding nucleotide regions of non-synonymous SNPs in the stress-responsive genes were analyzed using Pfam software [76] to determine the presence of functional domains/protein families within the genes. Amino acid sequences of such functional domain carrying stress responsive genes were analyzed further using the I-TASSER automated web server [77,78] for prediction of *ab intio* three dimensional secondary protein structure and active catalytic domain binding sites with ligands. The high quality protein model of correct topology and protein-ligand complex active binding sites was selected based on high confidence (C≥−1.5) and binding site (BS≥0.5) cut-off scores.

### Analysis of nucleotide diversity, population genetic structure and association

The SNP loci validated in the stress-responsive gene sequences across 91 diverse rice genotypes were aligned using CLUSTALW multiple sequence alignment tool in MEGA [79] 4.0 and results exported in meg format. The meg files were analyzed further using *DNaSp* 4.0 [80] to calculate polymorphism information content (PIC) [81] and genetic distances (D_ij_) across the genotypes. The genotypic data were used in a model-based program “STRUCTURE” [82] to determine population structure using admixture and correlated allele frequency with a burn-in of 50,000 iterations and run length of 100,000. A model-based clustering algorithm was applied in STRUCTURE that identified sub-population groups with distinctive allele frequencies and individual rice genotypes placed into K clusters. Of the many alternative structure models that varied for independent runs of the algorithm, K (population number) = 4 representing better relationships among the *indica*, aromatic, *japonica* and *aus*/wild rice genotype groups at α value less than 0.2 was selected. Twenty independent runs with K = 4 were carried out to determine the consistency of results obtained. Various population genetic parameters including fixation of different SNP loci in different sub-populations and their efficiency for detecting genetic variability (F_ST_) and degree of admixture within and between groups were estimated.

Genotyping data of validated SNP loci in stress-responsive rice genes and phenotypic information of 91 rice genotypes belonging to three different ecosystems namely, upland, medium land and lowland (see Additional file 6) were analyzed using the TASSEL software tool [83,84] to identify genes and novel alleles/SNPs associated with ecological differentiation in rice. A General Linear Model (GLM) considering the multiple levels of ancestry coefficient data (Q matrix) as obtained above in population genetic structure at population number (K) = 4 and relative kinship (K) matrix estimated from SPAGeDi 1.2 [85] were used to measure the two important parameters of trait association namely, P_adj_marker (significant association of SNPs in the genes with trait) and marker R_square (magnitude of association/correlation explained by the SNPs in the genes with traits). The GLM trait association model was permuted 1,000 times to optimize threshold significance level for association analysis. The SNP loci in the stress-responsive genes showing high degree of association with ecological adaptation in a set of rice genotypes at significant cut-off P_adj value ≤0.05 (with 95% confidence) and R^2^ value ≥0.90 were selected for further analysis.

## Competing interests

The authors declare that they have no competing intersets.

## Authors’ contributions

SKP conducted validation and genotyping of SNPs in stress-responsive rice genes, data analysis and drafted the manuscript. MM was involved in validation and genotyping of SNPs. AKS and NKS participated in selection of rice germplasm lines and also helped in drafting the manuscript. TM designed the study, guided data analysis and interpretation, participated in drafting and correcting the manuscript and gave the final approval of the version to be published. All authors have read and approved the final manuscript.

## Supplementary Material

Additional file 1 Validation and genotyping of 384 SNPs present in important abiotic and biotic stress-responsive rice genes across a representative set of 91 rice genotypes. Click here for file

Additional file 2 Chromosome-wise distribution of SNPs validated through GoldenGate genotyping assay. Click here for file

Additional file 3 **A non-synonymous SNP validated in a gene containing leucine-rich repeat (LRR) domain (LOC_Os04g19750) showing differentiation between eight known blast resistant *indica *and 30 blast susceptible *indica *rice genotypes.** Click here for file

Additional file 4 Optimization of number of sub-populations (K value) varying from K = 2 to K = 5 to determine the best possible population structure for 91 rice genotypes. Click here for file

Additional file 5 **Pair-wise estimates of genetic variance (F**_**ST**_**) among four O. sativa sub-populations.** Click here for file

Additional file 6 **Domesticated and wild *Oryza *genotypes used in the study and their inferred ancestry coefficients in population genetic structure analysis.** Click here for file

Additional file 7 Graphical genotyping using 325 SNP loci validated through Illumina GoldenGate assay across 91 rice genotypes based on their ascending order of physical location (bp) on 12 rice chromosomes giving allele sharing maps of individual rice genotypes. Click here for file

Additional file 8 **Genomic constitution of rice genotypes based on introgression of *indica *and *japonica *alleles on the 12 rice chromosomes.** Click here for file

Additional file 9 Graphical genotyping of 12 Tripura Medicinal rice chromosomes. Click here for file
