# Supplementary material for: SNPs in stress-responsive rice genes: validation, genotyping, functional relevance and population structure
Source: BMC Genomics. 2012 Aug 25;13:426. doi: 10.1186/1471-2164-13-426 (PMC3562522; doi:10.1186/1471-2164-13-426)
Supplement: Additional file 2 — Chromosome-wise distribution of SNPs validated through GoldenGate genotyping assay. [file 1471-2164-13-426-S2.doc]

**Additional file 2: Chromosome-wise distribution of SNPs validated through GoldenGate assay**

| **Chromosomes** | **No. of SNP loci**  **analyzed** | **No. of SNP loci successfully genotyped** | **No. of SNP loci showing polymorphism** | **Percent polymorphism** |
| --- | --- | --- | --- | --- |
| 1 | 50 | 48 | 43 | 86 |
| 2 | 40 | 39 | 36 | 90 |
| 3 | 18 | 18 | 18 | 100 |
| 4 | 45 | 43 | 40 | 89 |
| 5 | 33 | 30 | 28 | 85 |
| 6 | 32 | 29 | 26 | 81 |
| 7 | 36 | 33 | 28 | 78 |
| 8 | 26 | 24 | 23 | 88 |
| 9 | 30 | 28 | 23 | 77 |
| 10 | 28 | 26 | 21 | 75 |
| 11 | 19 | 18 | 18 | 95 |
| 12 | 27 | 26 | 21 | 78 |
| **Total** | **384** | **362** | **325** | **85** |
