# Supplementary material for: SNPs in stress-responsive rice genes: validation, genotyping, functional relevance and population structure
Source: BMC Genomics. 2012 Aug 25;13:426. doi: 10.1186/1471-2164-13-426 (PMC3562522; doi:10.1186/1471-2164-13-426)

**Additional file 3: A non-synonymous SNP validated in a gene containing leucine-rich repeat (LRR) domain (LOC_Os04g19750) showing differentiation between eight known Blast resistant *indica* and 30 Blast susceptible *indica* rice genotypes. Missense transitional substitution of the second nucleotide ‘T’ in the triplet codon GTC of eight upland *indica* coding for aminoacid valine by nucleotide ‘C’ resulted in formation of a new triplet codon GCC encoding alanine in lowland *indica* group which might produce a functionally different protein. Arrow indicates the non-synonymous SNP site.**

**Upland *indica***

**Lowland *indica***


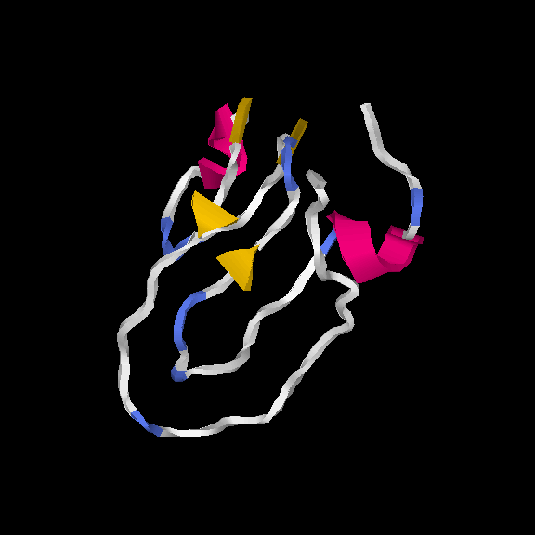

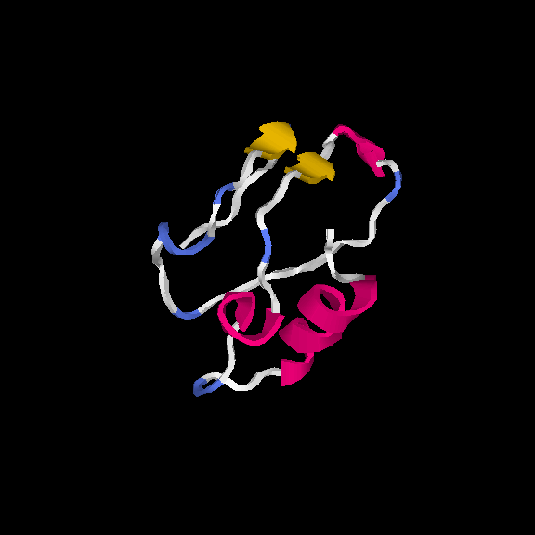

Supplement: Additional file 3 — A non-synonymous SNP validated in a gene containing leucine-rich repeat (LRR) domain (LOC_Os04g19750) showing differentiation between eight known blast resistant indica and 30 blast susceptible indica rice genotypes. [file 1471-2164-13-426-S3.doc]
