# Supplementary material for: SNPs in stress-responsive rice genes: validation, genotyping, functional relevance and population structure
Source: BMC Genomics. 2012 Aug 25;13:426. doi: 10.1186/1471-2164-13-426 (PMC3562522; doi:10.1186/1471-2164-13-426)
Supplement: Additional file 4 — Optimization of number of sub-populations (K value) varying from K = 2 to K = 5 to determine the best possible population structure for 91 rice genotypes. [file 1471-2164-13-426-S4.doc]

**Additional file 4: Optimization of number of sub-populations (K value) varying from K = 2 to K = 5 to determine the best possible population structure for 91 rice genotypes**


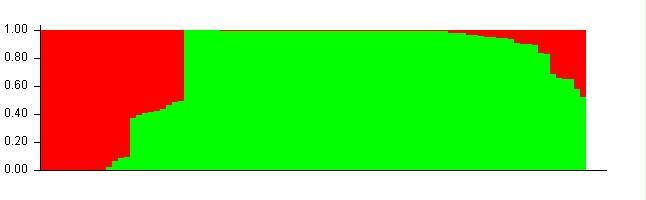

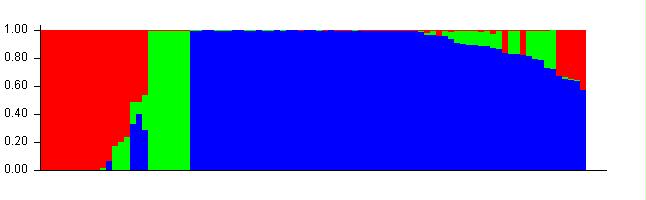


**K = 2**

**K = 3**

**K = 5**

**K = 4**

**Aromatics**

***japonica***

***indica***

***aus*/wild species**
