# Supplementary material for: SNPs in stress-responsive rice genes: validation, genotyping, functional relevance and population structure
Source: BMC Genomics. 2012 Aug 25;13:426. doi: 10.1186/1471-2164-13-426 (PMC3562522; doi:10.1186/1471-2164-13-426)
Supplement: Additional file 5 — Pair-wise estimates of genetic variance (FST) among four O. sativa sub-populations. [file 1471-2164-13-426-S5.doc]

**Additional file 5: Pair-wise estimates of genetic variance (F_ST_) among four *O. sativa* sub-populations**

| **Genotype groups** | **Domesticated *O. sativa* genotype groups** | | | |
| --- | --- | --- | --- | --- |
|  | **Long-grained aromatics** | **Short-grained aromatics** | ***indica* (including possible *aus* types)** | ***japonica*** |
| **Long-grained aromatics** | - | 0.50 | 0.48 | 0.31 |
| **Short-grained aromatics** | 0.52 | - | 0.54 | 0.37 |
| ***indica* (including possible *aus* types)** | 0.51 | 0.57 | - | 0.92 |
| ***japonica*** | 0.20 | 0.35 | 0.90 | - |

F_ST_ differs from zero (p < 10^-3^) with confidence interval ±0.0005 or narrower
