# Supplementary material for: SNPs in stress-responsive rice genes: validation, genotyping, functional relevance and population structure
Source: BMC Genomics. 2012 Aug 25;13:426. doi: 10.1186/1471-2164-13-426 (PMC3562522; doi:10.1186/1471-2164-13-426)
Supplement: Additional file 6 — Domesticated and wild Oryza genotypes used in the study and their inferred ancestry coefficients in population genetic structure analysis. [file 1471-2164-13-426-S6.doc]

**Additional file 6: Domesticated and wild *Oryza* genotypes used in the study and their inferred ancestry coefficients in population genetic structure analysis**

| **Genotypes used** | **Group** | **Eco-system** | **Inferred ancestry among *O. sativa* and wild cultivars** | | | |
| --- | --- | --- | --- | --- | --- | --- |
|  |  |  | **Aromatic** | ***japonica*** | ***indica*** | **Wild** |
| Taraori Basmati | Traditional long-grained Basmati | Lowland | 0.999 | 0.005 | 0.002 | 0.001 |
| Basmati-370 | Traditional long-grained Basmati | Lowland | 0.869 | 0.005 | 0.002 | 0.131 |
| Basmati 386 | Traditional long-grained Basmati | Lowland | 0.999 | 0.005 | 0.002 | 0.001 |
| Type3/Dehradun Basmati | Traditional long-grained Basmati | Lowland | 0.996 | 0.005 | 0.002 | 0.003 |
| CSR30 | Improved high-yielding long-grained Basmati | Lowland | 0.999 | 0.005 | 0.002 | 0.001 |
| Super Basmati | Improved high-yielding long-grained Basmati | Lowland | 0.999 | 0.005 | 0.002 | 0.001 |
| Shah Pasand | Improved high-yielding long-grained Basmati | Lowland | 0.678 | 0.082 | 0.002 | 0.239 |
| Pusa Sungadh5 | Improved high-yielding long-grained Basmati | Lowland | 0.849 | 0.151 | 0.002 | 0.001 |
| Hasan Sarai | Improved high-yielding long-grained Basmati | Lowland | 0.814 | 0.185 | 0.002 | 0.001 |
| Seond Basmati | Improved high-yielding long-grained Basmati | Lowland | 0.526 | 0.001 | 0.328 | 0.145 |
| Sona Mahsuri | Improved high-yielding long-grained Basmati | Lowland | 0.002 | 0.005 | 0.999 | 0.001 |
| Improved Pusa Basmati 1 | Improved high-yielding long-grained Basmati | Lowland | 0.388 | 0.005 | 0.611 | 0.001 |
| Pusa1121 | Improved high-yielding long-grained Basmati | Lowland | 0.564 | 0.005 | 0.300 | 0.135 |
| Pusa Basmati 1 | Improved high-yielding long-grained Basmati | Lowland | 0.462 | 0.005 | 0.537 | 0.001 |
| Haryana Basmati | Improved high-yielding long-grained Basmati | Lowland | 0.387 | 0.005 | 0.606 | 0.007 |
| Kasturi | Improved high-yielding long-grained Basmati | Lowland | 0.396 | 0.005 | 0.600 | 0.004 |
| Kalanamak1 | Traditional short-grained aromatics | Lowland | 0.871 | 0.128 | 0.001 | 0.001 |
| Bindli | Traditional short-grained aromatics | Lowland | 0.668 | 0.33 | 0.001 | 0.002 |
| Sonasal | Traditional short-grained aromatics | Lowland | 0.598 | 0.322 | 0.079 | 0.001 |
| Nipponbare | Temperate *japonica* | Lowland | 0.006 | 0.999 | 0.001 | 0.001 |
| Taipei309 | Tropical *japonica* | Lowland | 0.005 | 0.999 | 0.002 | 0.001 |
| Tripura Medicinal Rice | *indica* | Lowland | 0.178 | 0.538 | 0.004 | 0.28 |
| Sathi | *indica* | Lowland | 0.001 | 0.001 | 0.999 | 0.001 |
| Heera | *indica* | Upland | 0.001 | 0.001 | 0.998 | 0.001 |
| Narendra Dhan118 | *indica* | Upland | 0.024 | 0.002 | 0.868 | 0.106 |
| Neela | *indica* | Upland | 0.001 | 0.001 | 0.999 | 0.001 |
| ADT-37 | *indica* | Lowland | 0.003 | 0.001 | 0.911 | 0.085 |
| Rasi | *indica* | Upland | 0.001 | 0.001 | 0.728 | 0.27 |
| Annada | *indica* | Upland | 0.001 | 0.002 | 0.992 | 0.005 |
| Ratna | *indica* | Upland | 0.001 | 0.001 | 0.999 | 0.001 |
| IR36 | *indica* | Lowland | 0.002 | 0.001 | 0.994 | 0.004 |
| IR20 | *indica* | Lowland | 0.001 | 0.001 | 0.998 | 0.001 |
| Malviya Dhan-36 | *indica* | Lowland | 0.001 | 0.001 | 0.998 | 0.001 |
| Phalguna | *indica* | Lowland | 0.001 | 0.001 | 0.999 | 0.001 |
| Kranti | *indica* | Lowland | 0.001 | 0.001 | 0.999 | 0.001 |
| Lunisree | *indica* | Lowland | 0.002 | 0.001 | 0.759 | 0.239 |
| Hei-Bao | *indica* | Medium/lowland | 0.002 | 0.004 | 0.881 | 0.113 |
| Nagina-22 | *indica* | Upland | 0.008 | 0.001 | 0.001 | 0.991 |
| Pusa44 | *indica* | Lowland | 0.022 | 0.001 | 0.963 | 0.013 |
| Kalinga-III | *indica* | Upland | 0.001 | 0.001 | 0.999 | 0.001 |
| Aditya | *indica* | Upland | 0.001 | 0.001 | 0.785 | 0.213 |
| Jhum Khasa | *indica* | Upland | 0.003 | 0.001 | 0.001 | 0.996 |
| TKM-6 | *indica* | Lowland | 0.001 | 0.002 | 0.994 | 0.002 |
| Pant Dhan-12 | *indica* | Lowland | 0.001 | 0.002 | 0.996 | 0.002 |
| Narendra Dhan-359 | *indica* | Upland | 0.001 | 0.002 | 0.999 | 0.002 |
| Jaya | *indica* | Lowland | 0.001 | 0.002 | 0.999 | 0.002 |
| Pechi Badam | *indica* | Upland | 0.016 | 0.002 | 0.001 | 0.982 |
| Golmalati | *indica* | Upland | 0.001 | 0.002 | 0.001 | 0.998 |
| Tetep | *indica* | Lowland | 0.001 | 0.007 | 0.781 | 0.211 |
| PR-106 | *indica* | Lowland | 0.001 | 0.002 | 0.999 | 0.001 |
| ADT- 43 | *indica* | Lowland | 0.001 | 0.001 | 0.91 | 0.088 |
| Intan | *indica* | Lowland | 0.253 | 0.068 | 0.678 | 0.001 |
| Pokkali | *indica* | Lowland | 0.001 | 0.001 | 0.999 | 0.001 |
| Samba Mahsuri | *indica* | Lowland | 0.001 | 0.001 | 0.999 | 0.001 |
| Rajendra | *indica* | Medium/lowland | 0.001 | 0.001 | 0.827 | 0.173 |
| BirsaDhan | *indica* | Upland | 0.001 | 0.001 | 0.999 | 0.001 |
| Divya | *indica* | Lowland | 0.001 | 0.001 | 0.998 | 0.001 |
| Swarna | *indica* | Lowland | 0.002 | 0.001 | 0.993 | 0.004 |
| Vijetha | *indica* | Medium/lowland | 0.002 | 0.001 | 0.999 | 0.001 |
| CSR36 | *indica* | Lowland | 0.002 | 0.001 | 0.998 | 0.001 |
| PusaNPT11 | *indica* | Medium/lowland | 0.046 | 0.338 | 0.356 | 0.26 |
| Chaitanya | *indica* | Lowland | 0.001 | 0.002 | 0.998 | 0.001 |
| Red Triveni | *indica* | Upland | 0.009 | 0.001 | 0.892 | 0.098 |
| Sharbati | *indica* | Lowland | 0.001 | 0.001 | 0.999 | 0.001 |
| IR64 | *indica* | Lowland | 0.001 | 0.001 | 0.998 | 0.001 |
| IR24 | *indica* | Lowland | 0.001 | 0.005 | 0.871 | 0.123 |
| Urbashi | *indica* | Lowland | 0.001 | 0.002 | 0.935 | 0.064 |
| Nilagiri | *indica* | Upland | 0.001 | 0.002 | 0.999 | 0.001 |
| Shankar | *indica* | Medium/lowland | 0.002 | 0.002 | 0.912 | 0.086 |
| Pratap | *indica* | Medium/lowland | 0.001 | 0.002 | 0.999 | 0.001 |
| Khandagiri | *indica* | Upland | 0.001 | 0.002 | 0.933 | 0.066 |
| Subhadra | *indica* | Upland | 0.001 | 0.002 | 0.999 | 0.001 |
| Samanta | *indica* | Upland | 0.001 | 0.002 | 0.999 | 0.001 |
| Pathara | *indica* | Upland | 0.004 | 0.002 | 0.993 | 0.003 |
| Sarthi | *indica* | Medium/lowland | 0.041 | 0.001 | 0.956 | 0.002 |
| Satabdi | *indica* | Medium/lowland | 0.192 | 0.012 | 0.807 | 0.001 |
| Tapaswini | *indica* | Medium/lowland | 0.001 | 0.001 | 0.998 | 0.001 |
| Udaygiri | *indica* | Medium/lowland | 0.005 | 0.001 | 0.002 | 0.993 |
| Jagabandhu | *indica* | Lowland | 0.001 | 0.002 | 0.998 | 0.001 |
| Birupa | *indica* | Medium/lowland | 0.001 | 0.002 | 0.707 | 0.292 |
| Ananga | *indica* | Lowland | 0.178 | 0.002 | 0.821 | 0.011 |
| Bhanja | *indica* | Lowland | 0.001 | 0.002 | 0.997 | 0.011 |
| Daya | *indica* | Medium/lowland | 0.001 | 0.001 | 0.999 | 0.012 |
| Gajapathi | *indica* | Medium/lowland | 0.001 | 0.001 | 0.998 | 0.012 |
| Badami | *indica* | Upland | 0.006 | 0.001 | 0.845 | 0.148 |
| Kalyani-II | *indica* | Upland | 0.001 | 0.003 | 0.993 | 0.003 |
| Karjat-1 | *indica* | Medium/lowland | 0.03 | 0.001 | 0.947 | 0.022 |
| Konark | *indica* | Medium/lowland | 0.001 | 0.001 | 0.826 | 0.172 |
| IRBB60 | *indica* | Medium/lowland | 0.001 | 0.018 | 0.979 | 0.003 |
| *Oryza rufipogon* | Wild | Lowland | 0.001 | 0.263 | 0.186 | 0.550 |
| *Oryza nivara* | Wild | Lowland | 0.001 | 0.255 | 0.187 | 0.556 |

Yellow highlights indicate the maximum inferred ancestry coefficient of 91 rice genotypes that led to their correspondence with one model-based rice sub-population as determined by STRUCTURE
