# Supplementary material for: SNPs in stress-responsive rice genes: validation, genotyping, functional relevance and population structure
Source: BMC Genomics. 2012 Aug 25;13:426. doi: 10.1186/1471-2164-13-426 (PMC3562522; doi:10.1186/1471-2164-13-426)
Supplement: Additional file 7 — Graphical genotyping using 325 SNP loci validated through Illumina GoldenGate assay across 91 rice genotypes based on their ascending order of physical location (bp) on 12 rice chromosomes giving allele sharing maps of individual rice genotypes. [file 1471-2164-13-426-S7.doc]

**Additional file 7: Graphical genotyping using 325 SNP loci validated through Illumina GoldenGate assay across 91 rice genotypes based on their ascending order of physical location (bp) on 12 rice chromosomes giving allele sharing maps of individual rice genotypes. The discriminated allele types A (homozygous for allele A, *japonica* Nipponbare type), B (homozygous for allele B, *indica* 93-11 type) and H (heterozygous AB) are marked with red, blue and grey colors, respectively.**


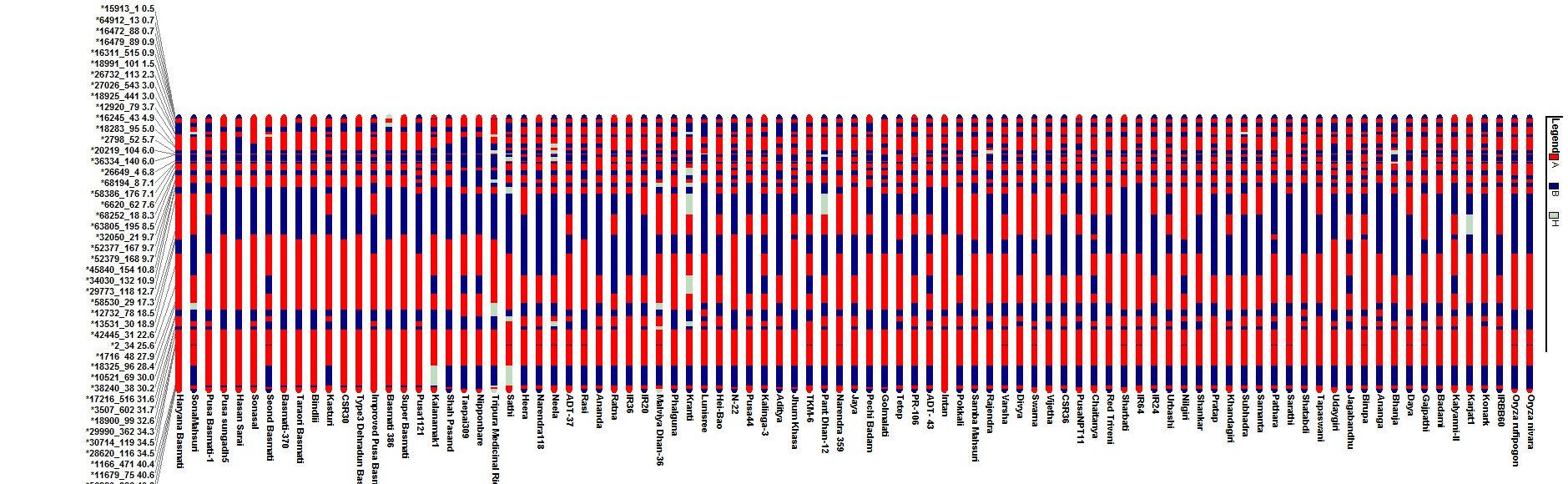


**Chromosome 1**


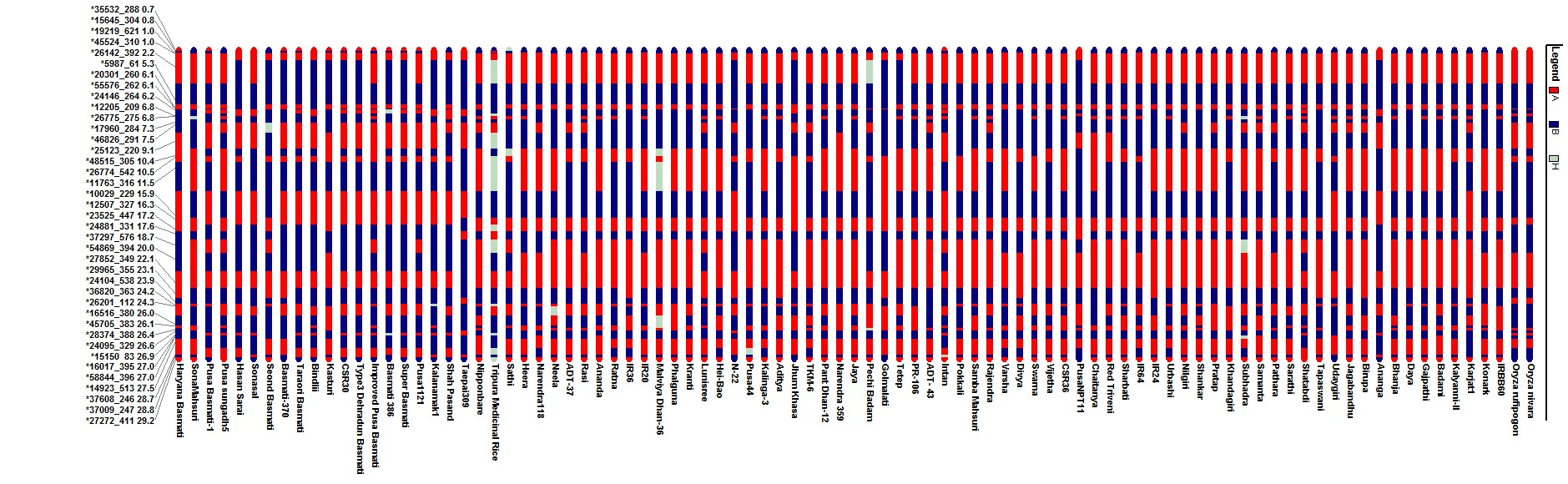


**Chromosome 2**


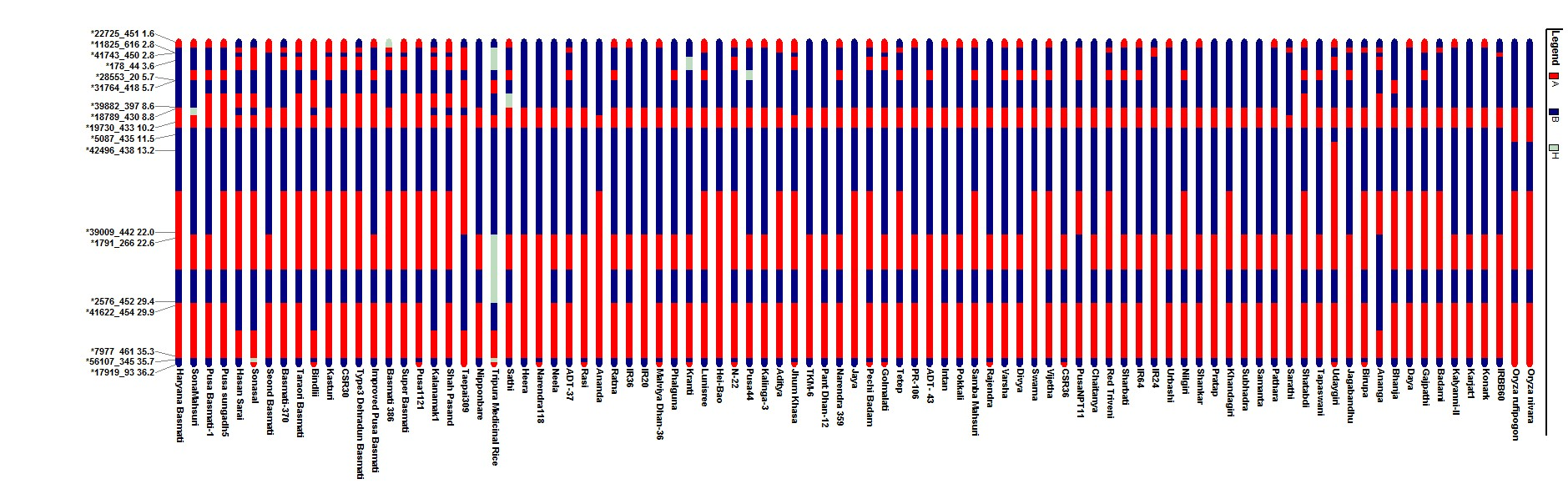


**Chromosome 3**


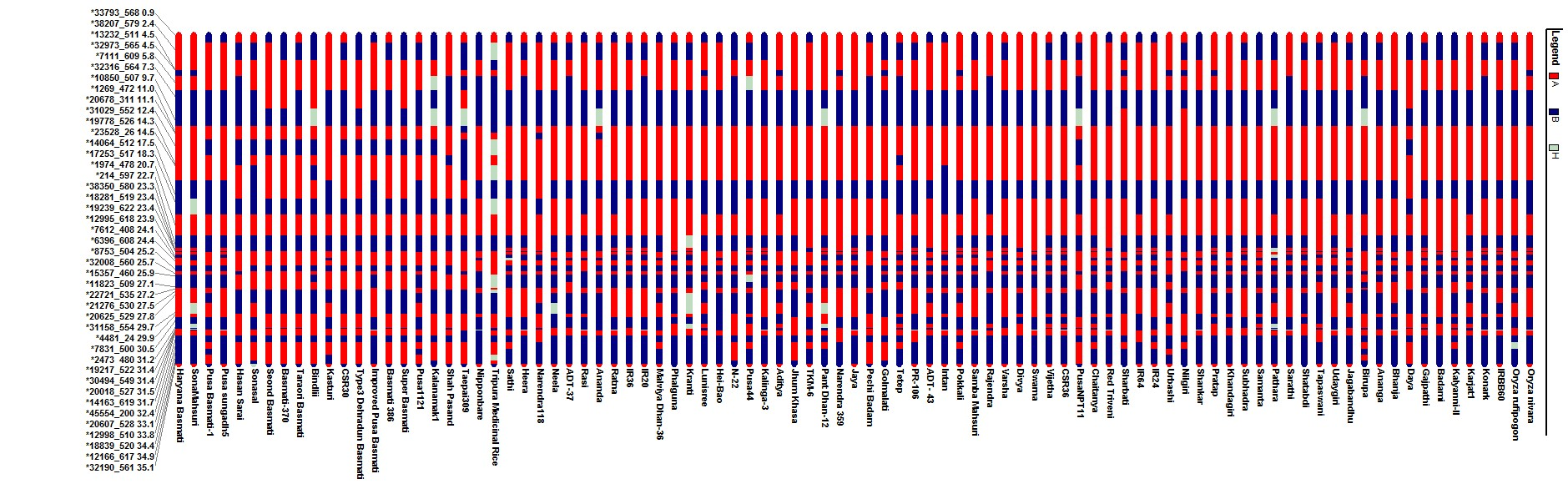


**Chromosome 4**


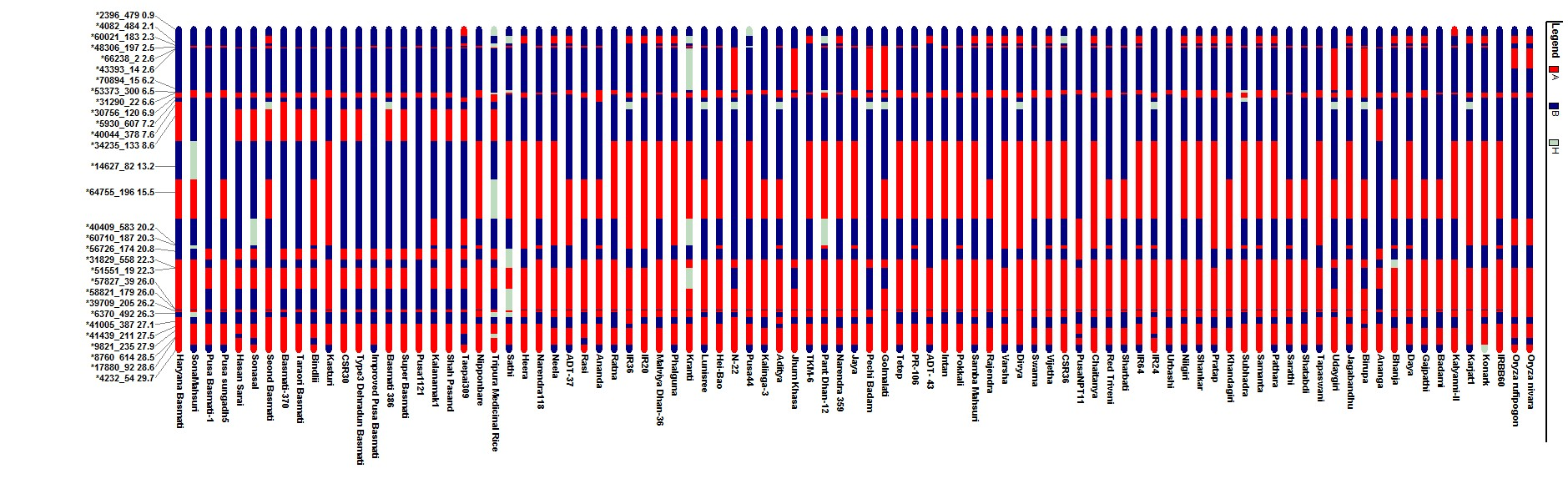


**Chromosome 5**


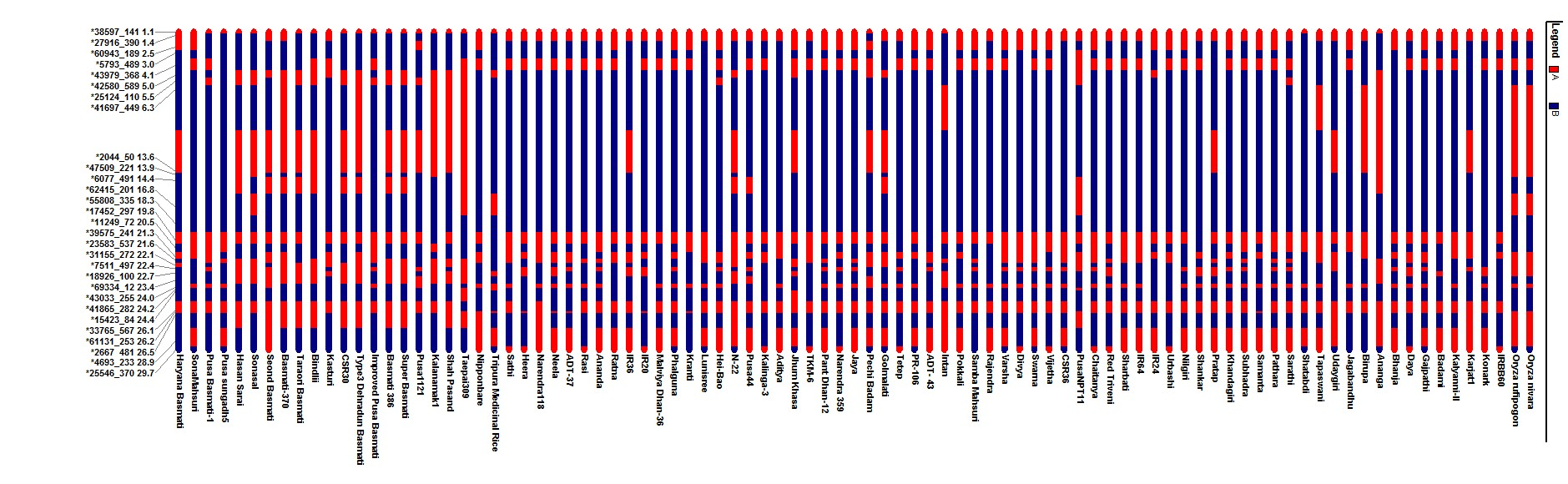


**Chromosome 6**


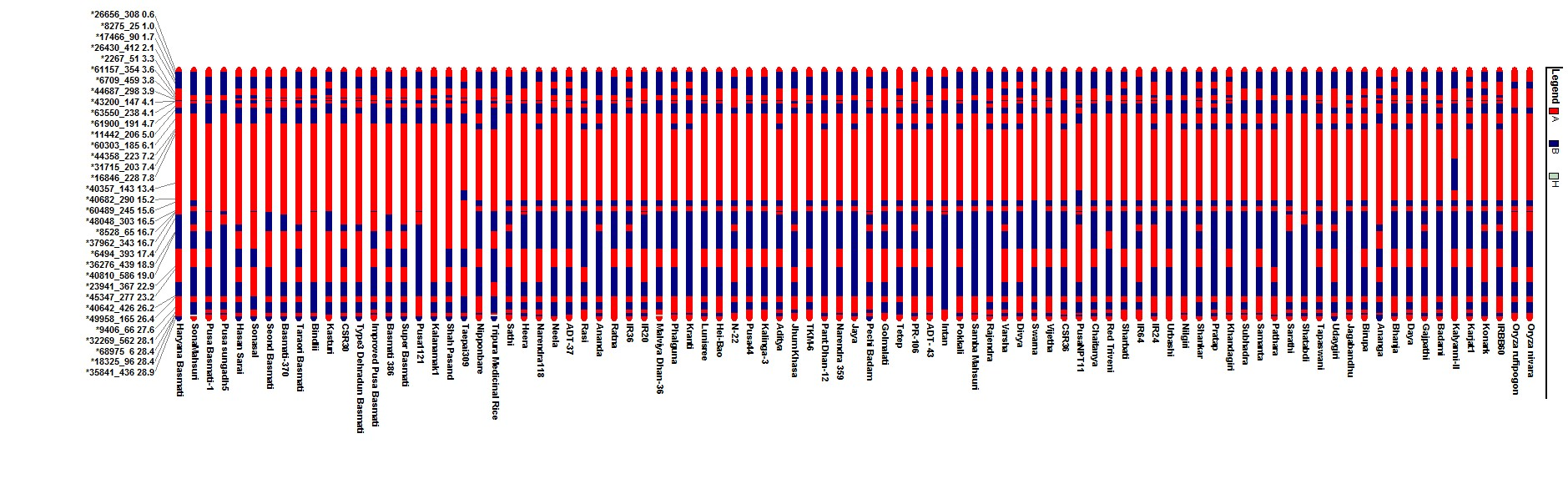


**Chromosome 7**


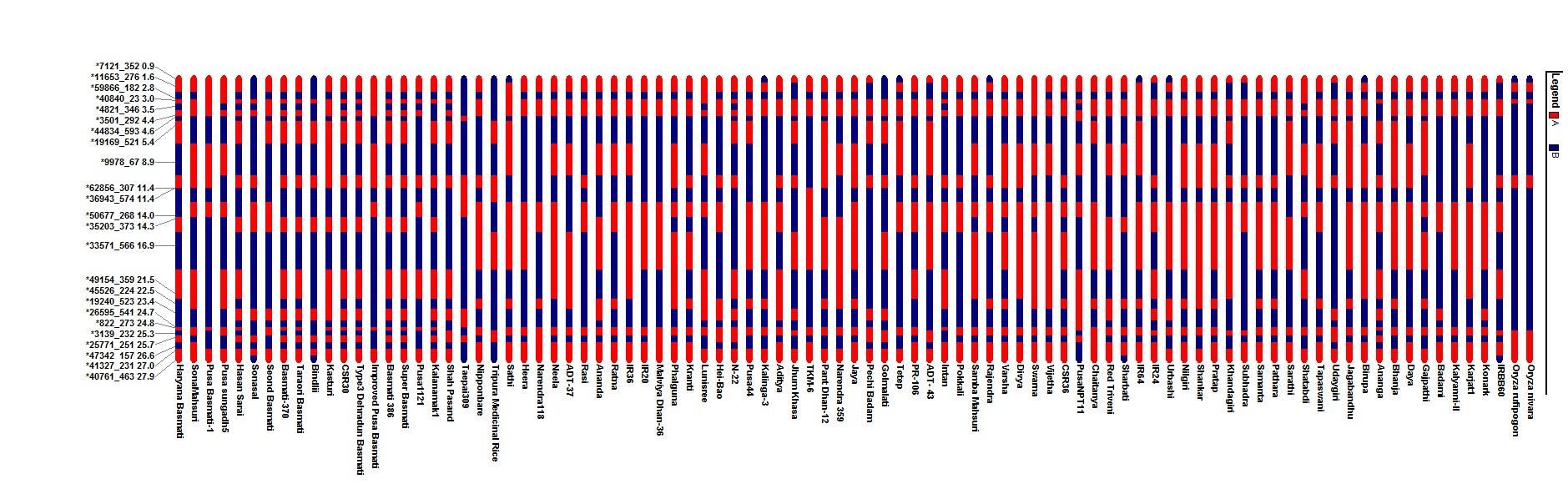


**Chromosome 8**


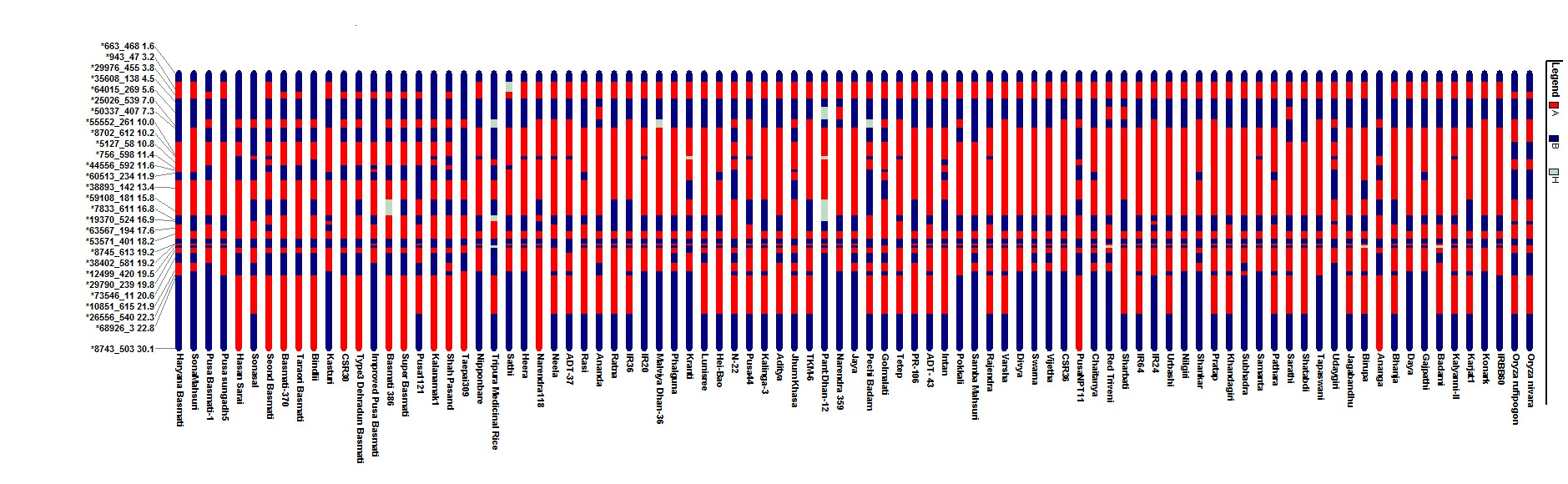


**Chromosome 9**


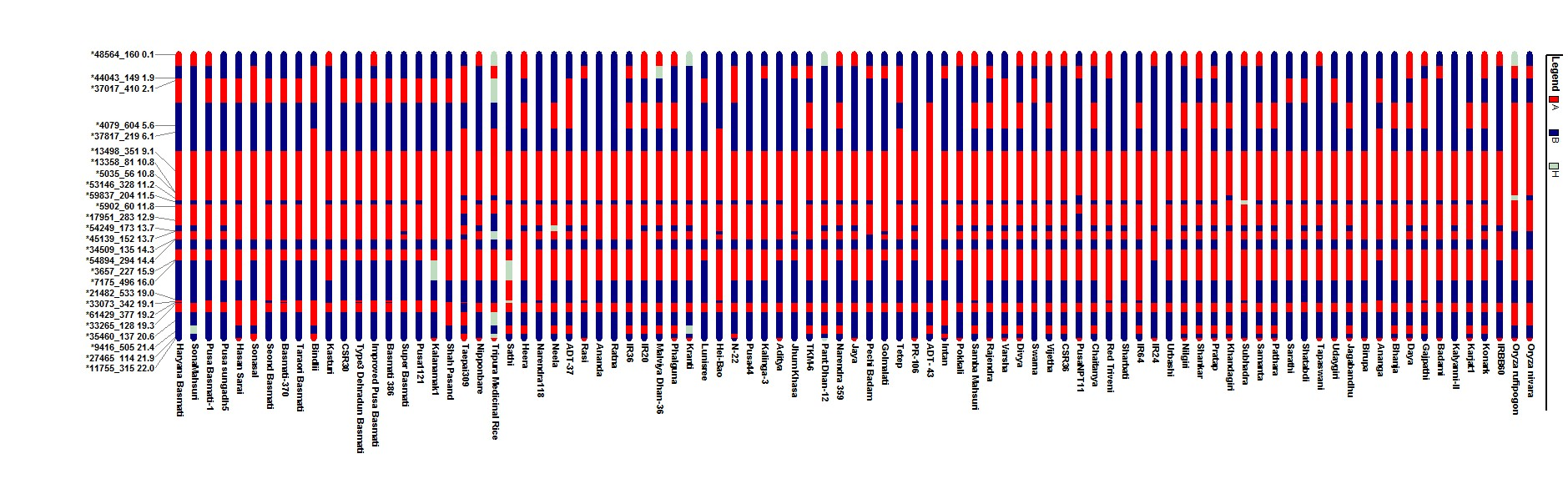


**Chromosome 10**


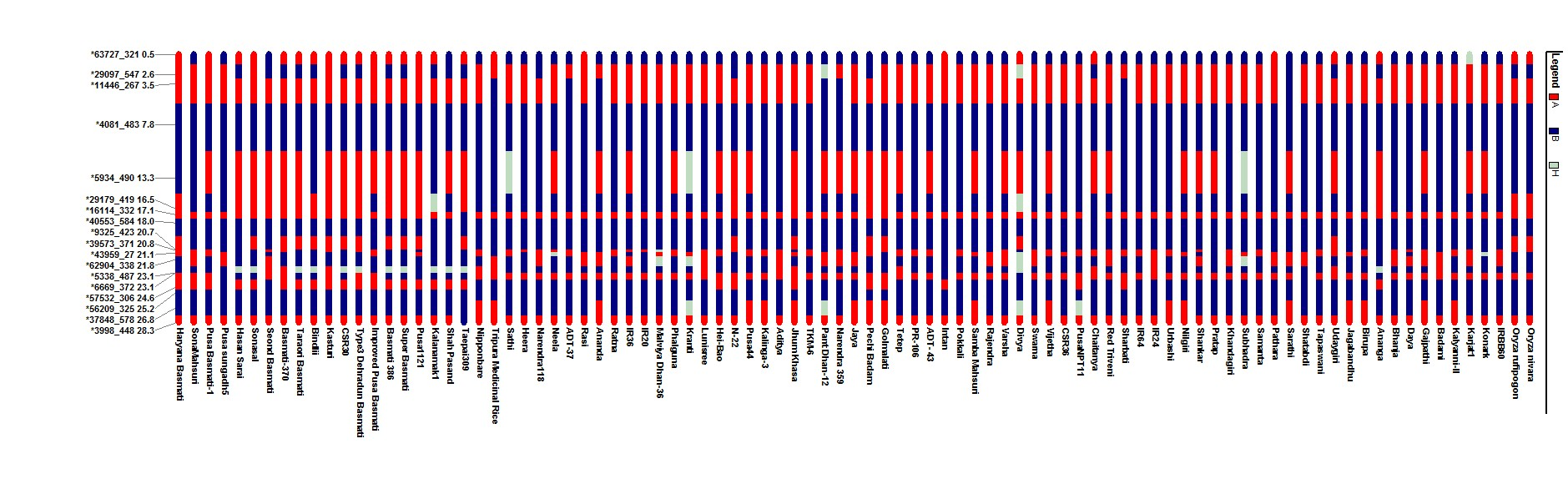


**Chromosome 11**


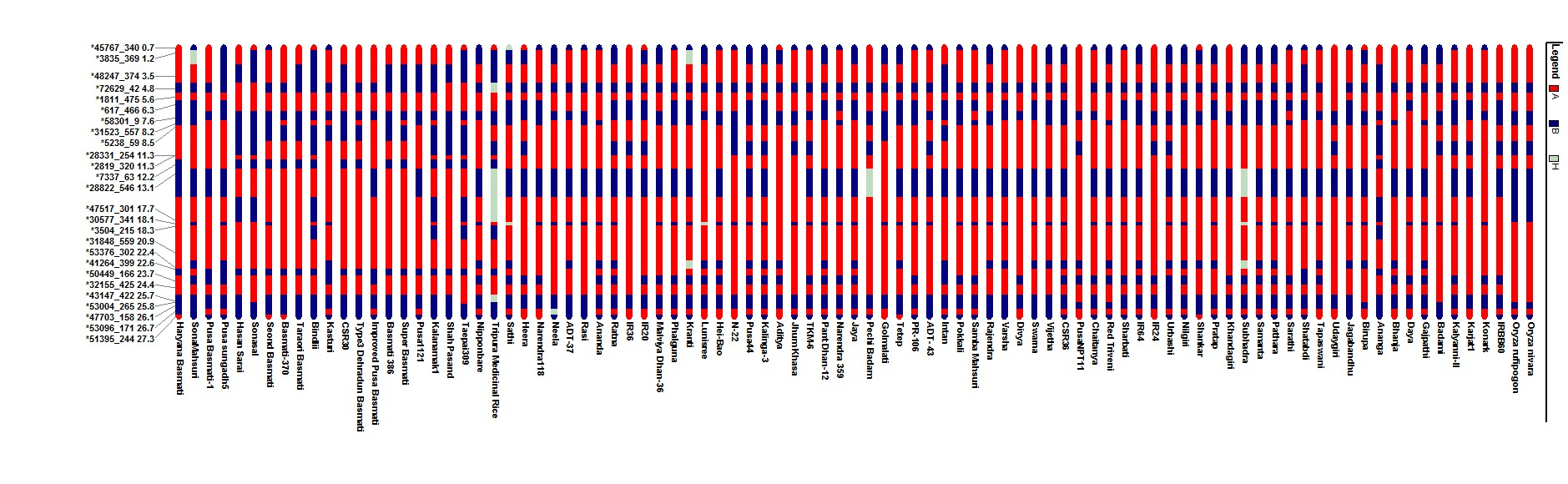


**Chromosome 12**
