# Supplementary material for: SNPs in stress-responsive rice genes: validation, genotyping, functional relevance and population structure
Source: BMC Genomics. 2012 Aug 25;13:426. doi: 10.1186/1471-2164-13-426 (PMC3562522; doi:10.1186/1471-2164-13-426)
Supplement: Additional file 8 — Genomic constitution of rice genotypes based on introgression of indica and japonica alleles on the 12 rice chromosomes. [file 1471-2164-13-426-S8.doc]

**Additional file 8: Genomic constitution of rice genotypes based on introgression of *indica* and *japonica* alleles on the 12 rice chromosomes**

| **Chromosomes** | **Introgression of *indica* and *japonica*** | | | |
| --- | --- | --- | --- | --- |
|  | **Proportion (%) of introgression of *japonica*** | **Proportion (%) of introgression of *indica*** | **Range (mean) of introgression frequency/ number of recombination** | **Chromosomal region showing maximum introgression frequency** |
| 1 | 57.0 | 42.0 | 16 to 30 (23.4) | Short-arm |
| 2 | 49.4 | 49.7 | 12 to 28 (20.2) | Both short and long-arm |
| 3 | 45.3 | 54.1 | 4 to 12 (7.8) | Both short and long-arm |
| 4 | 52.8 | 45.8 | 15 to 27 (20.2) | Long-arm |
| 5 | 45.0 | 53.4 | 8 to 21 (13.6) | Long-arm |
| 6 | 31.8 | 68.2 | 7 to 17 (11.8) | Long-arm |
| 7 | 59.6 | 40.1 | 14 to 31 (21.9) | Both short and long-arm |
| 8 | 49.8 | 50.2 | 6 to 20 (12.2) | Both short and long-arm |
| 9 | 54.1 | 45.5 | 8 to 17 (12.5) | Long-arm |
| 10 | 52.4 | 46.9 | 6 to 17 (12.0) | Long-arm |
| 11 | 37.0 | 61.3 | 6 to 13 (9.9) | Long-arm |
| 12 | 63.3 | 35.9 | 8 to 18 (13.2) | Both short and long-arm |
