# Supplementary material for: SNPs in stress-responsive rice genes: validation, genotyping, functional relevance and population structure
Source: BMC Genomics. 2012 Aug 25;13:426. doi: 10.1186/1471-2164-13-426 (PMC3562522; doi:10.1186/1471-2164-13-426)
Supplement: Additional file 9 — Graphical genotyping of 12 Tripura Medicinal rice chromosomes. [file 1471-2164-13-426-S9.doc]

**1**

**2**

**3**

**4**

**5**

**6**

**7**

**8**

**9**

**10**

**11**

**12**

**Additional file 9: Graphical genotyping of 12 Tripura Medicinal rice chromosomes. Blue and red colour codes for regions of introgression from *indica* and *japonica*, respectively and grey colour indicates the heterozygous regions.**
